# Retinal neurodegeneration as accelerated ocular aging: a review of nutritional and metabolic supplements

**DOI:** 10.3389/fragi.2026.1912337

**Published:** 2026-10-12

**Authors:** Seyed Mohamadmehdi Moshtaghion, Maryam Shayan, Sarvesh Ramakrishnan, Andrew Ham, Ashkan Shafaei Gharehkoshan, Michael G. Morley, Nazlee Zebardast, Francisco Javier Díaz-Corrales, Enrique Rodríguez de la Rúa, Deeba Husain, Lucia Sobrin, Tobias Elze, David S. Friedman, Mohammad Eslami

**Affiliations:** 1 Massachusetts Eye and Ear Infirmary, Boston, MA, United States; 2 Department of Integrative Pathophysiology and Therapies, Andalusian Molecular Biology and Regenerative Medicine Centre (CABIMER), Seville, Spain; 3 Department of Ophthalmology, Harvard Medical School, Boston, MA, United States; 4 University of Tulsa, Tulsa, OK, United States; 5 Harvard Medical School, Boston, MA, United States; 6 Saint Louis University, St. Louis, MO, United States; 7 Department of Ophthalmology, Virgen Macarena University Hospital, Sevilla, Spain

**Keywords:** age-related macular degeneration, diabetic retinopathy, glaucoma, mitochondrial dysfunction, neuroprotection, nicotinamide adenine dinucleotide (NAD+), retinal aging

## Abstract

Retinal neurodegeneration is a major cause of irreversible vision loss and a shared feature of age-related macular degeneration (AMD), diabetic retinopathy (DR), and glaucoma (GL). Increasing evidence suggests that these conditions share important pathways with retinal aging, including mitochondrial dysfunction, metabolic insufficiency, redox imbalance, chronic low-grade inflammation, and progressive neuronal vulnerability. While antioxidant and micronutrient supplementation, most notably the Age-Related Eye Disease Study (AREDS) formulations, has demonstrated benefit in selected AMD populations, emerging metabolic and nutraceutical strategies targeting aging-related pathways are of unproven benefit. In this review, following a brief overview of the current landscape of investigated supplements, we synthesize preclinical and clinical evidence for a group of metabolic supplements, including nicotinamide adenine dinucleotide (NAD^+^) boosters, citicoline, pyruvate, and omega-3 fatty acids, that directly modulate mitochondrial bioenergetics, cellular redox state, membrane integrity, and neuroinflammatory signaling—pathways that are recognized hallmarks of biological aging and are particularly relevant to aging-related processes implicated in retinal neurodegeneration. Using a structured literature and clinical trial search, we summarize mechanistic insights and translational findings across AMD, DR, and glaucoma, emphasizing convergence on core pathways linking retinal neurodegeneration and ocular aging. Overall, current evidence supports biological plausibility and early functional or biomarker signals for these agents, particularly in glaucoma and diabetic retinal disease, although long-term efficacy remains unproven. Framing retinal disease at the intersection of neurodegeneration and aging biology, this review highlights metabolic supplementation as a promising but evolving strategy for preserving retinal function.

## 1 Introduction

Retinal neurodegeneration represents a pressing public health challenge, given its substantial contribution to irreversible vision loss worldwide. With global populations aging rapidly, diseases such as age-related macular degeneration (AMD), diabetic retinopathy (DR), and glaucoma (GL) are projected to rise, threatening quality of life and increasing socioeconomic burdens (Bourne et al., 2024; Fleckenstein et al., 2024; Lundeen et al., 2023). Together, AMD, DR, and glaucoma account for the majority of irreversible blindness globally, each representing distinct yet interconnected pathways of retinal damage. These three conditions together reflect the spectrum of neurodegenerative, vascular, and metabolic mechanisms underlying retinal pathology. Their shared risk factors including aging, metabolic syndrome, oxidative stress, and inflammation, highlight converging biological pathways that make them model diseases for studying vision-related aging and neurodegeneration. Importantly, preventive strategies are urgently needed, as once these diseases are established, therapeutic options are often limited, only partially effective, or associated with substantial economic and societal costs (Bourne et al., 2024; Fleckenstein et al., 2024; Lundeen et al., 2023).

Growing evidence suggests that nutritional supplementation may contribute to retinal neuroprotection, reflecting the retina’s exceptionally high metabolic demand and vulnerability to oxidative stress. Consistent with this interest, a substantial body of literature and an increasing number of clinical trials have investigated dietary supplements across age-related macular degeneration, glaucoma, and diabetic retinopathy. As summarized in Figures 1, 2, we identified 51 registered clinical trials and 89 published studies evaluating a wide range of nutritional interventions for these conditions, underscoring both the clinical relevance and the growing momentum of this research area.

**FIGURE 1:**
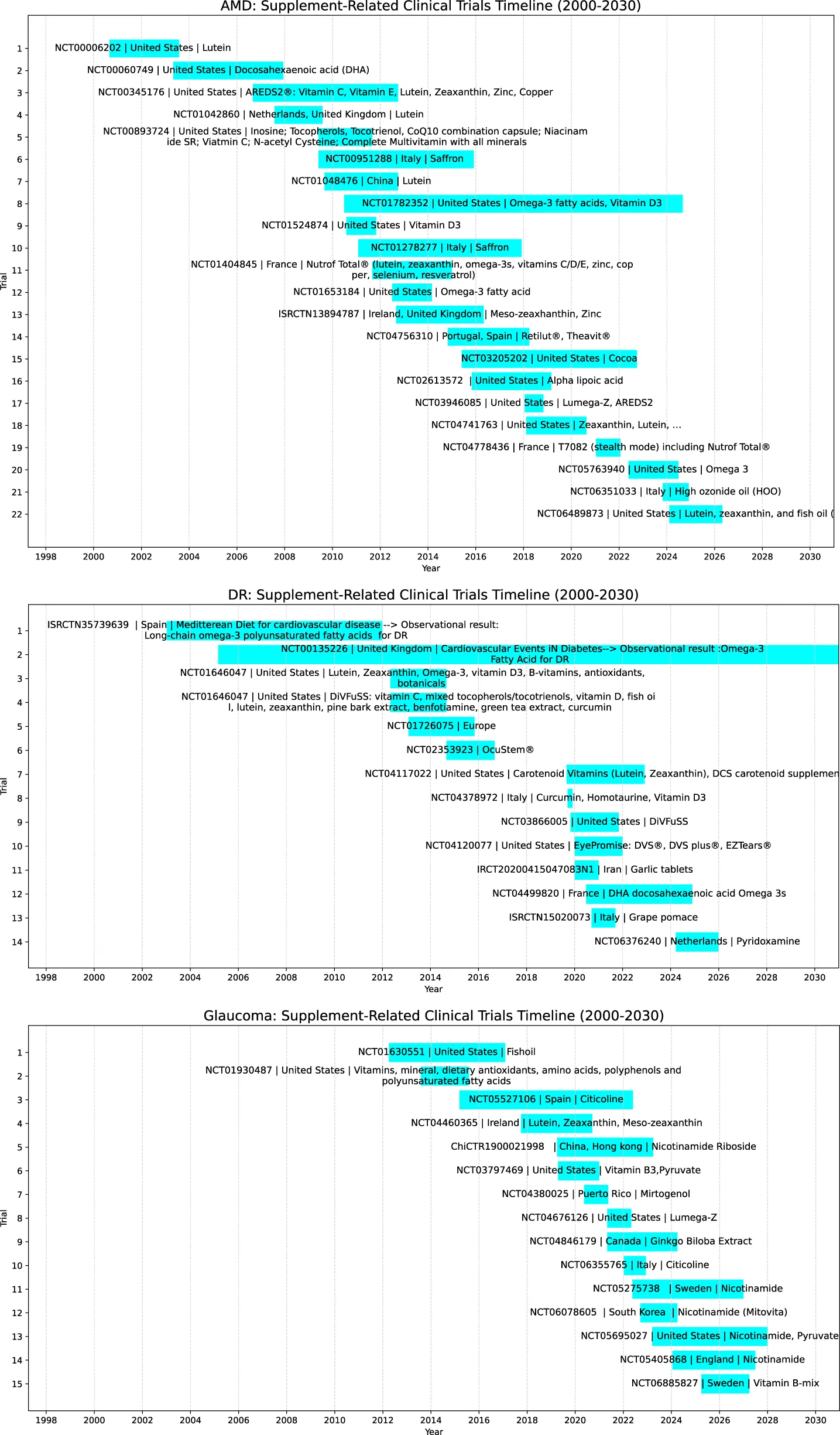
Timeline of supplement-related clinical trials (2000–2030). Cyan bars indicate trial duration and the text shows trial registered number, country and supplement details.

**FIGURE 2:**
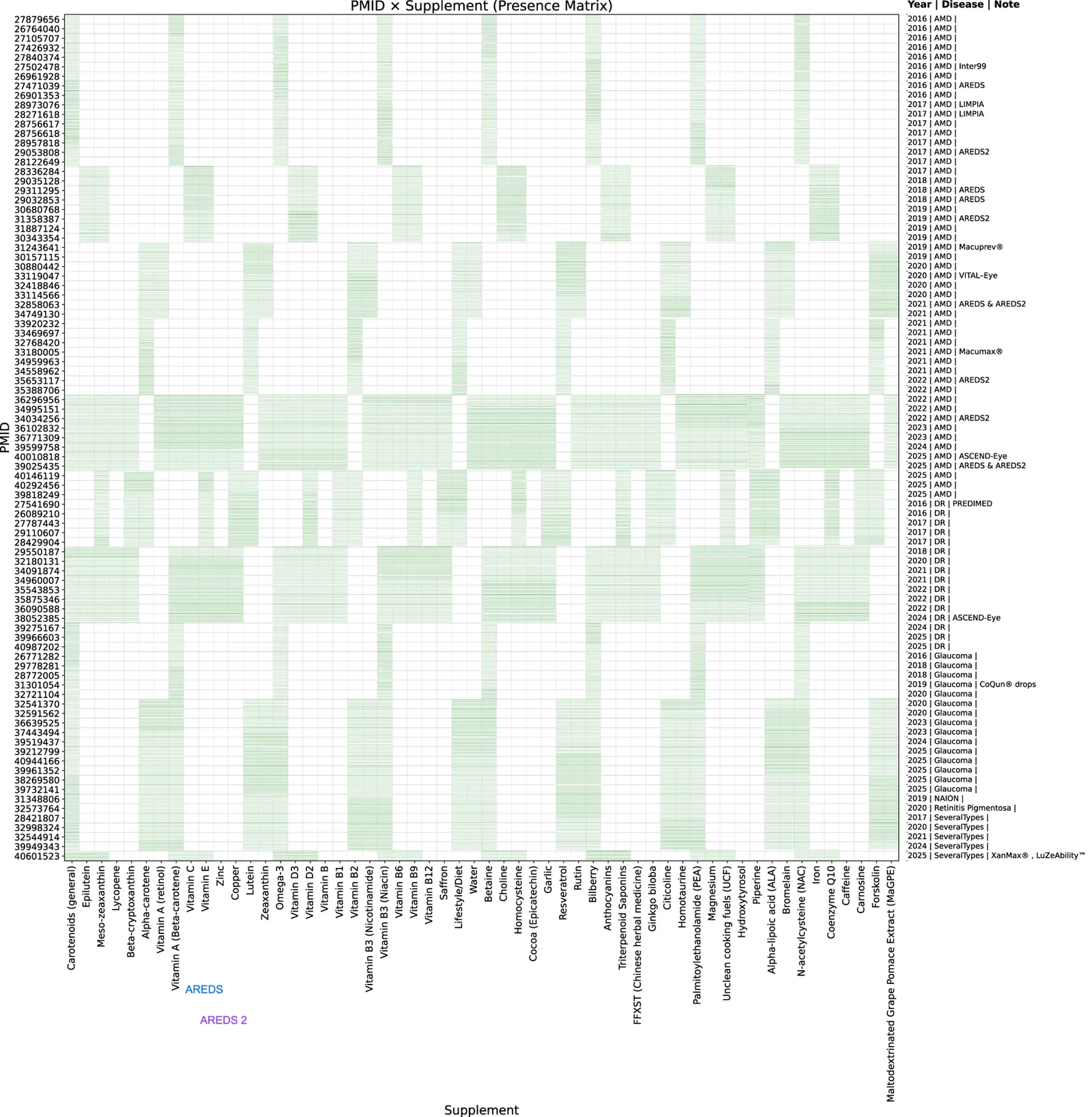
The presence matrix, showing papers and corresponding discussed/investigated supplements.

The original Age-Related Eye Disease Study (AREDS) demonstrated that a formulation containing antioxidant vitamins and zinc reduced the risk of progression to advanced AMD in individuals at elevated risk. AREDS2 subsequently evaluated modifications to this formulation, including the addition of lutein and zeaxanthin and omega-3 fatty acids and the removal of beta-carotene in certain formulations, refining the composition and safety of AREDS-based supplementation (Bonds et al., 2014; Chew et al., 2013; Gunvant Davey et al., 2020; Keenan et al., 2020; Study Details NCT00345176, 2026).

### 1.1 Scope and conceptual framework

Unlike prior reviews that primarily summarize supplements within individual diseases, this review adopts a translational framework that conceptualizes retinal neurodegeneration within the context of accelerated ocular aging. AMD, DR, and glaucoma have distinct and multifactorial disease mechanisms; however, they also share convergent aging-related pathways, including mitochondrial dysfunction, oxidative stress, chronic inflammation, metabolic insufficiency, and progressive neuronal vulnerability. The organizing principle of this review is that AMD, DR, and glaucoma may be viewed within the framework of accelerated retinal aging, while recognizing their distinct disease-specific mechanisms (**Ocular Disease ↔ Aging ↔ Metabolic Supplements**) (Campello et al., 2021). This framework justifies evaluating supplements not only for disease-specific efficacy but also for their capacity to modulate biological hallmarks of aging as they occur in ocular tissue. Therefore, after a brief overview of the current supplement landscape, we focus on four specific supplements (NAD + boosters, citicoline, pyruvate, and omega-3 fatty acids) to examine their potential neuroprotective mechanisms.

This paper is organized as follows. The Introduction outlines the search strategy and reviews age-related and pathological changes in the retinal nervous system, highlighting key hallmarks of ocular aging and the concept of ocular biological age. Section 2 provides a brief overview of the current landscape of nutritional supplements investigated in retinal disease. Subsequent sections systematically examine the evidence supporting the neuroprotective effects of four selected metabolic supplements. In the Discussion, we address uncertainties and assess the level of confidence in the available evidence to inform clinician–patient discussions regarding nutritional interventions. A comprehensive list of registered clinical trials evaluating nutritional supplements in glaucoma, diabetic retinopathy, and age-related macular degeneration, together with their reported findings, is provided in the Appendix.

### 1.2 Retinal neurodegeneration

#### 1.2.1 Age-related changes in the retinal nervous system

Retinal neurons have exceptionally high metabolic demand and are highly susceptible to mitochondrial dysfunction, which worsens with aging as oxidative phosphorylation declines, reactive oxygen species (ROS) accumulate, and mitochondrial DNA mutations increase, leading to impaired ATP production, disrupted homeostasis, and heightened vulnerability of retinal ganglion cells, photoreceptors, and the retinal pigment epithelium (RPE) to degeneration. These bioenergetic deficits, coupled with oxidative stress from excess ROS and weakened antioxidant defenses, drive lipid, protein, and DNA damage, activate apoptotic pathways, and promote para-inflammation characterized by chronic low-grade microglial activation and pro-inflammatory cytokine release, ultimately accelerating neuro-retinal and neuro-vascular degeneration (Buonfiglio et al., 2024; Tong et al., 2022; Xu et al., 2025; Zhang et al., 2025).

Normal aging is characterized by a progressive reduction in retinal neuronal populations. Histological and *in vivo* imaging studies have consistently demonstrated that retinal ganglion cells (RGCs) decline at an annual rate of approximately 0.5%–0.6%, corresponding to a loss of 6,000–6,700 RGCs per year (Vilasboas-Campos et al., 2025). Photoreceptor attrition, particularly of rod cells, has also been noted, resulting in diminished scotopic sensitivity and increased threshold variability in perimetric assessments. The loss of these neuronal populations underpins the age-dependent decrease in spatial and contrast sensitivity observed clinically (Bell et al., 2020; Lee et al., 2022).

Quantitative optical coherence tomography (OCT) studies provide normative data documenting age-associated thinning of the retinal nerve fiber layer (RNFL), ganglion cell layer (GCL), and ganglion cell–inner plexiform layer (GCIPL). The peripapillary RNFL shows a mean annual thinning of 0.07–0.2μm, with pronounced losses in the temporal and inferior quadrants. GCIPL thickness also decreases linearly with age, correlating with cumulative loss of ganglion cell bodies. Standard automated perimetry and macular microperimetry reveal modest but consistent age-related declines in visual field sensitivity. These functional losses often become clinically appreciable after the age of 70, affecting both central and peripheral retinal function (Lee and Mackey, 2022; Sano et al., 2025; Vilares-Morgado et al., 2026).

The concept of ocular biological age refers to the assessment of molecular and cellular characteristics that reflect the cumulative impact of aging hallmarks on retinal and other ocular tissues. It is driven by biological aging, characterized by genomic instability, telomere attrition, epigenetic alterations, changes in DNA methylation, loss of proteostasis, mitochondrial dysfunction, cellular senescence, altered intercellular communication, stem cell exhaustion, and chronic inflammation (Min et al., 2024). Epigenetic clocks are among the most widely used tools to assess biological age, relying on DNA methylation patterns across the genome which shift with age and are intimately linked to processes like mitochondrial function, stem cell population dynamics, and nutrient sensing (Kabacik et al., 2022).

Recent advances highlight those epigenetic mechanisms, particularly DNA, RNA, and histone methylation, play central roles in retinal and ocular diseases (Pan et al., 2025; Xia et al., 2024; Xu et al., 2024). Methylation regulates gene expression without altering DNA sequences and serves as a key interface between environmental factors, metabolism, and genome function. Aberrant methylation patterns have been linked to retinal neurodegeneration, including DR, glaucoma, and AMD, where dysregulated methyltransferases (DNMTs) and demethylases (TETs) alter pathways governing inflammation, oxidative stress, and vascular remodeling.

In experimental and clinical studies, changes in ocular DNA methylation patterns, accumulation of senescent cells, mitochondrial dysfunction, and altered inflammatory responses have all been observed with advancing chronological age. Recent applications of omics technologies and deep learning have refined ocular aging clocks, enabling researchers to integrate DNA methylation, transcriptomic, proteomic, and imaging data to assess eye-specific biological aging (Wolf et al., 2023; Zhang et al., 2025). Such clocks can identify when the biological age of ocular tissues exceeds chronological age, a signal of accelerated ocular aging associated with increased risk for age-related diseases, including macular degeneration and glaucoma. Despite their predictive utility, ongoing research aims to correlate ocular clock values with clinical endpoints, explore how ocular-specific molecular hallmarks manifest differently from systemic aging, and identify interventions that can slow or reverse the biological aging of the eye (Eells, 2019; Kaarniranta et al., 2020; Moshtaghion et al., 2025; Wolf et al., 2023; Zhang et al., 2025).

#### 1.2.2 Glaucoma

Glaucoma comprises a heterogeneous group of progressive optic neuropathies characterized by degeneration of RGCs and their axons, leading to irreversible visual field loss. It is the second leading cause of blindness globally, with current estimates indicating that over 76 million individuals are affected. Its prevalence is expected to increase markedly with an aging population. Primary open-angle glaucoma is the most prevalent form, while primary angle-closure glaucoma is particularly significant among populations in East and Southeast Asia (Qi et al., 2025; Zhao et al., 2023). The supplement evidence discussed in this review pertains exclusively to open-angle glaucoma; no comparable data exist for angle-closure disease.

The principal risk factor for glaucoma is elevated intraocular pressure (IOP), which is the only modifiable factor for which established therapeutic interventions exist. Advanced age, genetic susceptibility, and a positive family history increase glaucoma risk. The genetic architecture is heterogeneous and varies across glaucoma phenotypes; for example, pathogenic variants in MYOC are associated with juvenile- and adult-onset open-angle glaucoma, OPTN variants have been implicated particularly in some forms of normal-tension and open-angle glaucoma, whereas CYP1B1 variants are most strongly associated with primary congenital and juvenile-onset glaucoma. Ethnicity is a critical determinant: individuals of African descent have a higher risk and earlier onset of open-angle glaucoma, whereas angle-closure forms predominate in Asian populations. Other factors, such as thin central corneal thickness and vascular comorbidities, also modulate susceptibility and progression (Bou Ghanem et al., 2024; Fahmideh et al., 2022; Jayaram et al., 2023; Stein et al., 2021).

Recent evidence suggests an association between systemic biological aging, estimated using DNA methylation–based “epigenetic clocks,” and glaucoma progression. In a retrospective study of patients with primary open-angle glaucoma (POAG), Medeiros et al. evaluated epigenetic age using DNA methylation profiles derived from blood samples and found greater epigenetic-age acceleration among fast *versus* slow glaucoma progressors. Specifically, each additional year of age acceleration measured using the Horvath clock was associated with 15% higher odds of fast progression (OR, 1.15; 95% CI, 1.07–1.23; P < 0.001). These findings demonstrate an association between systemic epigenetic-age acceleration and glaucoma progression but do not establish causality or validate an eye-specific aging clock (Medeiros et al., 2025; Song et al., 2025).

#### 1.2.3 Diabetic retinopathy (DR)

DR is a complex neurovascular complication of diabetes mellitus involving interconnected neuronal, glial, and microvascular dysfunction and represents a leading cause of vision impairment among working-age adults globally. The prevalence of DR is closely tied to the worldwide epidemic of diabetes, with recent meta-analyses estimating a global prevalence of any DR at approximately 35%, including proliferative forms and clinically significant macular edema. The risk of sight-threatening DR increases with both the duration and poor metabolic management of diabetes, making it a significant public health burden as the diabetic population exceeds 500 million worldwide. Historical cohorts reported a high prevalence of DR after approximately 20 years of diabetes, particularly in type 1 diabetes; however, contemporary risk varies substantially according to diabetes type and glycemic, blood-pressure, and lipid control. Managing Glycemic control, as measured by hemoglobin A1c, hypertension and hyperlipidemia shown to reduce risk in large interventional studies (Li G. et al., 2025; Teo et al., 2021; Wondmeneh and Mohammed, 2024).

#### 1.2.4 Age-related macular degeneration (AMD)

AMD is a progressive, degenerative condition of the central retina, primarily targeting the macula and representing the most common cause of irreversible central vision loss among individuals over 50 years of age globally. The prevalence of AMD is projected to increase substantially, with estimates suggesting nearly 300 million affected worldwide by 2040, making it a central public health concern as populations age. AMD is clinically categorized based on morphological changes and severity into early, intermediate, and advanced stages. Early AMD is marked by the accumulation of medium-sized drusen and minor pigmentary abnormalities, typically without significant visual symptoms. Intermediate AMD is characterized by larger, confluent drusen and pronounced RPE irregularities, which may begin to impair central vision. Advanced AMD manifests as either geographic atrophy, characterized by progressive loss of the RPE and photoreceptor cells in the macula, or neovascular (wet or exudative) AMD, which is caused by choroidal neovascularization and, in turn, leads to hemorrhage, fluid exudation, and fibrous scarring, rapidly compromising central vision (Christoforidis J et al., 2011; Cheung, 2025; Fleckenstein et al., 2021; Mitchell et al., 2018).

Major risk factors for AMD include advanced age, genetic predisposition (particularly variants in complement factor H (CFH) and ARMS2/HTRA1) and modifiable factors such as cigarette smoking which is consistently associated with a two-to threefold increased risk and faster disease progression, as well as hypertension, obesity, and high dietary fat intake. Chronic exposure to blue or ultraviolet light has been proposed as a potential environmental contributor based on experimental and epidemiologic observations; however, evidence establishing it as a major independent risk factor for AMD in humans remains limited and inconsistent. Cardiometabolic disease and a family history of AMD further exacerbate these risks (Hu et al., 2025; Jeong et al., 2025; Kong et al., 2025).

### 1.3 Review methodology

We performed a structured literature and clinical trial search to identify preclinical and clinical evidence on emerging supplements for ocular health. For the literature review, we searched PubMed/MEDLINE for studies published within the past 10 years using the following advance search terms:(supplement*[tiab] OR nutraceutical*[tiab] OR “dietary supplement*”[tiab] OR vitamin*[tiab] OR micronutrient*[tiab] OR antioxidant*[tiab] OR “omega-3”[tiab] OR PUFA[tiab]) AND (eye[tiab] OR ocular[tiab] OR retina*[tiab] OR retinal[tiab] OR vision[tiab] OR visual[tiab] OR ophthalm*[tiab]) AND (“Age-Related Macular Degeneration”[Mesh] OR “Diabetic Retinopathy”[Mesh] OR “Glaucoma”[Mesh] OR “macular degeneration”[tiab] OR “diabetic macular edema”[tiab] OR “diabetic retinopathy”[tiab] OR glaucoma[tiab]) AND (Clinical Trial[pt] OR Randomized Controlled Trial[pt] OR Meta-Analysis[pt]).

For the clinical trial search, studies were identified by selecting *Supplement* in the *Intervention/treatment* field and querying Diabetic Retinopathy, Age-Related Macular Degeneration, and Glaucoma separately in the *Condition/Disease* field, with results aggregated manually.

The study selection process is summarized using PRISMA-style flow diagrams in Appendix. Two reviewers independently screened the identified records. The PubMed/MEDLINE search resulted in 89 included articles. The ClinicalTrials.gov search identified 46 records, of which 39 met the eligibility criteria. In addition, 12 eligible trials identified through the literature search but not retrieved by the ClinicalTrials.gov search were added, resulting in a total of 51 clinical trials.

To support a deeper discussion of the underlying mechanisms, we supplemented this core set with additional references, including preclinical evidence, that were not part of the original structured search.

## 2 Overview of nutritional supplements

Nutritional and nutraceutical interventions have been extensively explored as adjunctive strategies across major ophthalmic diseases, particularly age-related macular degeneration (AMD), diabetic retinopathy (DR), diabetic macular edema (DME), and glaucoma. Overall, the literature reflects a heterogeneous but maturing landscape, characterized by modest sample sizes, diverse formulations, frequent reliance on intermediate biomarkers, and disease-specific variability in observed benefit. Figures 1, 2 show a comprehensive list of these studies.

Figure 1 presents the temporal distribution of supplement-related clinical trials across three major ophthalmic conditions, including age-related macular degeneration (AMD), diabetic retinopathy (DR), and glaucoma, from 2000 to 2030. The timelines illustrate a gradual increase in clinical research activity over the past 2 decades, with a noticeable acceleration after 2010. In AMD, trials predominantly focus on carotenoids (e.g., lutein, zeaxanthin), omega-3 fatty acids, and antioxidant formulations such as AREDS-based supplements. DR-related trials show a broader diversity of interventions, including omega-3 fatty acids, vitamins, polyphenols, and plant-derived compounds, reflecting the multifactorial nature of the disease. In glaucoma, more recent trials emphasize neuroprotective and metabolic supplements, such as citicoline, nicotinamide, and mitochondrial-targeting compounds. Overall, the figure highlights both the growing interest in nutritional interventions and the heterogeneity of supplement strategies across different ophthalmic diseases.

Figure 2 illustrates the presence matrix linking individual studies (PMIDs) to the supplements investigated across AMD, diabetic retinopathy (DR), glaucoma, and related conditions. Each row represents a study, while columns correspond to specific supplements or supplement classes, with highlighted cells indicating inclusion within the study. The matrix reveals a strong concentration of research around carotenoids (e.g., lutein, zeaxanthin), antioxidant vitamins (C, E), and zinc, largely driven by formulations aligned with AREDS and AREDS2. In contrast, more recent and less standardized interventions, such as nicotinamide, citicoline, alpha-lipoic acid, and polyphenol-based compounds, appear more sparsely and heterogeneously distributed across studies, particularly in DR and glaucoma. The figure also highlights the frequent co-occurrence of multiple supplements within single trials, reflecting combination-based therapeutic strategies rather than isolated interventions. Overall, the matrix underscores both the dominance of a few well-established formulations and the fragmented exploration of emerging supplements across ophthalmic research.

Considering Figure 2 and as summarized in Table 1, the current landscape of nutritional interventions is highly heterogeneous, spanning compounds with markedly different mechanisms, levels of evidence, and translational maturity. Importantly, only a subset of interventions demonstrates convergence across aging biology, retinal neurodegeneration, and emerging clinical evidence. Therefore, rather than comprehensively discussing all available supplements with equal emphasis, this review prioritizes compounds that provide mechanistic insight into shared pathways of retinal aging while showing meaningful translational potential. Consequently, after a brief overview on them, intentionally focuses more on nicotinamide adenine dinucleotide (NAD^+^) boosters, citicoline, pyruvate, and omega-3 fatty acids. This selection, which aligns with the recent trend in registered clinical trials, was guided by four considerations. 1) These agents converge mechanistically on core pathways underlying both retinal neurodegeneration and ocular aging, including mitochondrial dysfunction, metabolic insufficiency, redox imbalance, impaired proteostasis, and loss of neuronal membrane integrity processes shared across age-related macular degeneration, diabetic retinopathy, and glaucoma (Campello et al., 2021). 2) These compounds target biological hallmarks of aging that are particularly relevant to the retina, reduced mitochondrial resilience, altered lipid composition, and chronic low-grade inflammation, thereby linking systemic aging biology with tissue-specific neurodegeneration (Campello et al., 2021). 3) Each agent demonstrates a coherent translational trajectory spanning preclinical models, biomarker alterations, and early functional or interventional human evidence, distinguishing them from supplements supported primarily by observational or dietary associations. 4) Agents such as alpha-lipoic acid, palmitoylethanolamide, carotenoids, and polyphenol-based compounds were identified in our literature search but were not selected for focused review because they either lack a coherent translational trajectory across all three diseases, are primarily disease-specific in their evidence base (e.g., carotenoids in AMD *via* AREDS), or do not yet demonstrate modulation of core aging-related biological pathways beyond antioxidant activity.

**TABLE 1:** Major advances in metabolic ocular neuroprotection research.

Intervention	Primary mechanism	Major discovery	Evidence level	Translational readiness
NAD boosters/nicotinamide	Mitochondrial and metabolic support; RGC neuroprotection	Short-term improvement/signals in glaucoma; long-term preservation still under active trial	Early clinical/phase 2 signals	Low–moderate
Citicoline	Mitochondrial and metabolic support; RGC neuroprotection	Short-term improvement/signals in glaucoma; long-term preservation still under active trial	Early clinical/phase 2 signals	Low–moderate
Omega-3	Lipid signaling, anti-inflammatory/pro-resolving mediators	Observational AMD associations; AREDS2 did not show added AMD benefit; RCT evidence mixed by disease	Moderate but inconsistent	Moderate
Pyruvate	Energetic/metabolic support; glycolysis/mitochondrial rescue	Strong preclinical glaucoma rationale; human evidence mainly through nicotinamide + pyruvate trials	Preclinical to early clinical	Low
AREDS/AREDS2	Antioxidant + zinc support; lutein/zeaxanthin in AREDS2	Reduces progression from intermediate to advanced AMD by ∼25%; newer analysis supports benefit even in some late dry AMD cases	Strong clinical	High

Accordingly, this section provides a brief overview of the current landscape of nutritional supplements, followed by focused sections that examine the four selected supplements in greater detail. A comprehensive list of all identified supplements, along with corresponding studies and clinical trials, is presented in Figures 1, 2 and in the Appendix for completeness.

### 2.1 Age-related macular degeneration (AMD)

AMD remains the most intensively studied condition in the supplement literature. Carotenoids and macular pigments (lutein, zeaxanthin, meso-zeaxanthin) form the backbone of this field, largely motivated by their biological role in macular pigment formation and blue-light filtering. Multiple randomized trials demonstrate improvements in functional outcomes such as contrast sensitivity and glare sensitivity, as well as increases in macular pigment optical density (MPOD), particularly when bioavailability-optimized formulations are used (Choe et al., 2025; Gunvant Davey et al., 2020; Machida et al., 2020). However, results are not uniform; some trials report minimal MPOD changes across imaging modalities, lens status, and baseline AMD severity, underscoring measurement and population effects (Azar et al., 2017).

Multi-ingredient antioxidant formulations are common. A double-blind randomized trial of Macuprev®, a complex supplement containing carotenoids, vitamins, zinc, and botanical extracts, demonstrated improvement in macular electrophysiologic function (mfERG) in intermediate AMD without clear structural OCT changes (Parravano et al., 2019). While promising, such formulations limit mechanistic attribution.

Minerals, particularly zinc, show more consistent biomarker-level effects. Zinc acetate supplementation in early AMD patients with low baseline zinc improved oxidative stress markers (↓ malondialdehyde, ↑ superoxide dismutase) and reduced soft drusen area over 12 weeks (Mano et al., 2021). In contrast, iron supplementation raises safety concerns: *post hoc* analyses of large AMD treatment trials associate oral iron use with increased retinal and subretinal hemorrhage risk in neovascular AMD, particularly among hypertensive patients (Song et al., 2019).

Beyond antioxidants, alpha-lipoic acid (ALA) illustrates the mixed evidence typical of repurposed nutraceuticals. While an earlier randomized study reported improvements in vision-related quality of life in dry AMD, a later multicenter phase-2 trial found no benefit of oral ALA in slowing geographic atrophy progression or preserving visual acuity (Kim et al., 2020; Tao et al., 2016). Emerging approaches such as triterpenoid saponins, aimed at improving retinoid transport across Bruch’s membrane, demonstrated improved dark adaptation kinetics in a small proof-of-concept randomized trial, though confirmatory studies are needed (Lee et al., 2025).

### 2.2 Diabetic retinopathy (DR) and diabetic macular edema (DME)

Compared with AMD, the supplement landscape in DR and DME is newer but expanding. Several studies focus on oxidative stress modulation, reflecting its central role in diabetic microvascular damage. A randomized clinical trial showed that correcting vitamin D deficiency in patients with DME receiving intravitreal bevacizumab led to superior long-term functional and anatomical outcomes compared with vitamin-D-deficient patients who were not supplemented (Fekri et al., 2022).

Botanical and polyphenol-based interventions are gaining attention. A 6-month randomized placebo-controlled trial of maltodextrinated grape pomace extract demonstrated improvements in best-corrected visual acuity, reductions in central retinal thickness, stabilization of retinal perfusion, and significant decreases in oxidative stress biomarkers (dROMs and oxLDL) in non-proliferative DR (Schiano et al., 2024). Similarly, garlic supplementation showed modest but significant improvements in visual acuity and intraocular pressure in DME over a short follow-up period (Afarid et al., 2022).

Longer-term antioxidant vitamin combinations (B, C, and E) have been associated with slower DR development and improved biochemical profiles in patients with type 2 diabetes (Pramanik et al., 2022), though such studies often combine supplements with systemic metabolic management, complicating causal inference (Lafuente et al., 2017).

### 2.3 Glaucoma and ocular hypertension

In glaucoma, supplements are primarily explored as adjuncts to IOP-lowering therapy, targeting vascular dysregulation and retinal ganglion cell dysfunction. Palmitoylethanolamide (PEA) is among the more consistently supported compounds. A randomized crossover trial demonstrated improvements in pattern electroretinogram amplitude, modest IOP reduction, and quality-of-life gains in stable glaucoma patients (Rossi et al., 2020). These findings are reinforced by a recent systematic review and meta-analysis reporting significant IOP-lowering effects across multiple clinical studies (Crupi et al., 2025).

Antioxidant and vasomodulatory supplements yield mixed results. An antioxidant formulation containing Ginkgo biloba improved retrobulbar and retinal blood flow metrics in a randomized crossover trial, yet a later OCT-angiography-based clinical trial found no clinically meaningful improvement in macular or peripapillary perfusion with ginkgo extract (Harris et al., 2018; Hodgson et al., 2025). Multi-ingredient oral supplements combining forskolin, homotaurine, carnosine, folate, B vitamins, and magnesium have demonstrated reductions in IOP and improvements in electrophysiologic and functional endpoints over 12 months, though sample sizes remain small (Mutolo et al., 2016).

Of note, some agents occasionally grouped with supplements, such as Mexidol, are better classified as pharmacologic antioxidants rather than dietary supplements, despite being discussed in the context of neuroprotection (Egorov et al., 2025).

### 2.4 Conflicting and negative findings

The supplement literature in ophthalmology is not uniformly positive. While some compounds consistently improve functional or biochemical markers, true disease-modifying effects are rare, and several supplements show neutral or even adverse associations. Recognizing these opposing results strengthens the case for phenotype-specific, mechanism-driven, and safety-aware use, rather than broad, indiscriminate supplementation.

In the management of AMD, although carotenoids (lutein, zeaxanthin, meso-zeaxanthin) frequently improve contrast sensitivity and MPOD, structural or disease-modifying benefits are inconsistent: 1) No MPOD improvement despite supplementation: A multicenter, double-masked study using Visucam® imaging found no significant MPOD increase after 1 year of lutein (5 mg) and zeaxanthin (1 mg) supplementation, even in AMD eyes (Azar et al., 2017). Cataract status and imaging modality strongly influenced results, suggesting that measurement bias and optical confounders may explain discrepancies with other positive MPOD studies. 2) Functional gains without structural benefit: In the Macuprev® trial, mfERG function improved, but OCT structural parameters did not change (Parravano et al., 2019). This reinforces the idea that carotenoids may enhance neural efficiency or signal quality rather than halt degeneration. 3) Genotype–treatment controversy (AREDS/AREDS2): Early analyses suggested genotype-dependent harm or benefit from antioxidants and zinc (e.g., CFH/ARMS2 interactions) (Gunvant Davey et al., 2020; Seddon et al., 2016). However, independent reanalyzes failed to confirm clinically actionable genotype–treatment interactions, contradicting earlier claims (Assel et al., 2018; Seddon et al., 2016; Vavvas et al., 2018). This led to the consensus that genotyping should not guide supplement use in AMD.

Alpha-Lipoic Acid (ALA) serves as a clear example of initial clinical optimism eventually being met with null results in the context of age-related macular degeneration (AMD). Early findings provided a positive signal, suggesting that ALA could improve vision-related quality of life and boost antioxidant markers in patients with the dry form of the disease (Tao et al., 2016; Study Details NCT02613572, 2026). However, a subsequent and more definitive multicenter, randomized phase-2 trial concluded with a negative result; the study found no significant reduction in the progression of geographic atrophy and no measurable benefit to visual acuity over an 18-month period (Kim et al., 2020).

While some supplements are viewed as protective, others have demonstrated concerning or even harmful associations in the context of macular health. For instance, oral iron supplementation has been linked to an increased risk of retinal and subretinal hemorrhages in patients with neovascular AMD. This risk is particularly pronounced in hypertensive individuals and exhibits a clear dose-response relationship, suggesting that higher levels of iron may exacerbate vascular issues within the eye (Song et al., 2019). Similarly, dietary Vitamin A intake has shown unexpected results regarding early AMD; higher consumption was found to increase the risk of drusen formation, especially in those carrying CFH Y402H risk alleles (Munch et al., 2016). These findings challenge the common perception that fat-soluble vitamins are universally beneficial and highlight the importance of genetic context in nutritional supplementation.

In the management of glaucoma, Ginkgo biloba serves as a primary example of biomarker-outcome dissociation, where initial physiological improvements do not necessarily translate to long-term clinical success. Early research offered a positive outlook, demonstrating improved retrobulbar and retinal blood flow velocities after just 1 month of use (Harris et al., 2018). However, a more recent clinical trial utilizing optical coherence tomography angiography (OCT-A) provided a negative, or even opposing, result: researchers found no improvement—and actually observed a small reduction—in peripapillary perfusion density after 4 months of treatment (Hodgson et al., 2025).

Even among more promising glaucoma adjuncts, the results remain nuanced. Palmitoylethanolamide (PEA) has shown consistent ability to lower intraocular pressure (IOP) and improve pattern electroretinogram (PERG) readings (Crupi et al., 2025; Rossi et al., 2020); however, these effects are considered modest, and the compound remains an adjunctive therapy rather than a disease-modifying one. Furthermore, common exercise-related supplements can have conflicting impacts on ocular health; for instance, caffeine has been shown to acutely increase IOP, whereas beta-alanine does not, illustrating how widely used substances can transiently worsen ocular physiology in ways patients might not expect (Vera et al., 2024).

## 3 Nicotinamide adenine dinucleotide (NAD^+^)

NAD^+^-related supplementation represents one of the most mechanistically grounded candidate neuroprotective strategies in ophthalmology, especially for glaucoma. We identified seven trials investigating the NAD + boosters for glaucoma intervention (Leung et al., 2022; Study Details NCT06885827, 2026; Study Details NCT05405868, 2026; Study Details NCT05695027, 2026; Study Details NCT05275738, 2026; Study Details NCT03797469, 2026; Study Details NCT06078605, 2026). Nicotinamide stands out as a rare example where preclinical, mechanistic, and early clinical functional data align. In contrast, evidence in AMD and DR remains largely associative or preclinical, with no definitive disease-modifying trials to date.

NAD^+^ is an essential metabolic coenzyme that fuels mitochondrial oxidative phosphorylation and ATP generation and processes especially critical for the retina’s exceptionally high energy demand (Xie et al., 2020). Mammalian cells synthesize NAD^+^ through three canonical pathways.

1. *De novo* kynurenine pathway, which converts tryptophan into NAD^+^.
2. Preiss–Handler pathway, which utilizes nicotinic acid (NA).
3. Salvage pathway, which recycles nicotinamide (NAM) and nicotinamide riboside (NR) through nicotinamide mononucleotide (NMN) as the key intermediate (Bogan and Brenner, 2008; Lin and Apte, 2018; Verdin, 2015).

Among these, the salvage pathway provides ∼80% of NAD^+^ in most tissues, including the retina, making it the dominant source of cellular NAD^+^ (Lin and Apte, 2018). Its rate-limiting enzyme, NAMPT, converts NAM to NMN, while nicotinamide mononucleotide adenylyltransferases (NMNATs) catalyze the final step of NAD^+^ formation (Cantó et al., 2015). The three NMNAT isoforms have distinct subcellular roles.

- NMNAT1 (nuclear) maintains genome stability and supports DNA repair.
- NMNAT2 (cytosolic/Golgi) is neuron-enriched and essential for axonal maintenance; its loss triggers Wallerian degeneration.
- NMNAT3 (mitochondrial) sustains oxidative metabolism and normal mitochondrial function (Cimaglia et al., 2020).

Figure 3 outlines the major NAD^+^ biosynthetic routes, including kynurenine/*de novo*, Preiss–Handler, and salvage pathways and their relevance to AMD, DR and glaucoma.

**FIGURE 3:**
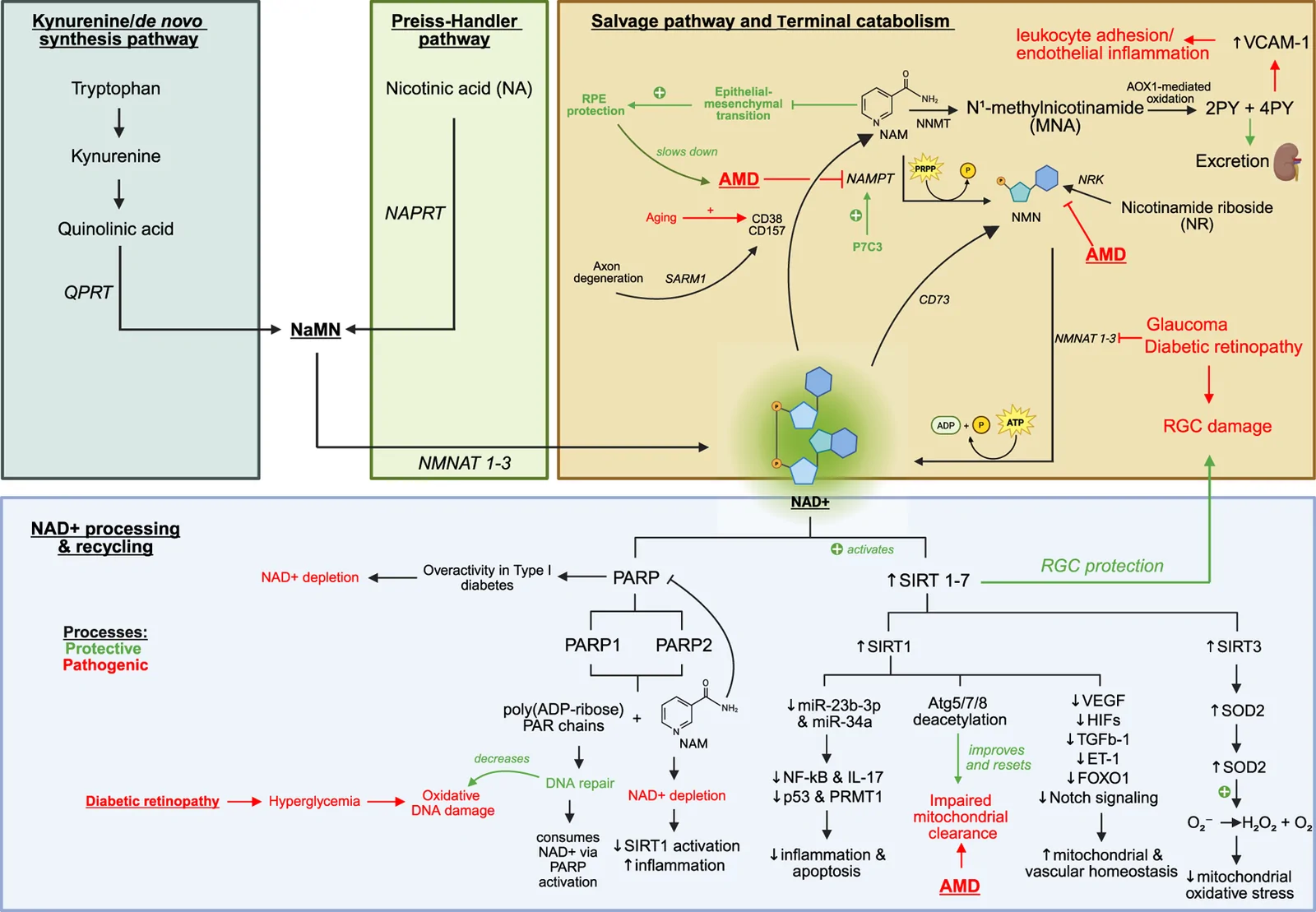
NAD^+^ biosynthetic pathways and their dysregulation in retinal neurodegenerative diseases. The figure outlines the major NAD^+^ biosynthetic routes—kynurenine/*de novo*, Preiss–Handler, salvage and terminal catabolism pathways—and their relevance to AMD, DR and glaucoma. The salvage pathway is most affected in retinal degeneration, where reduced NAMPT activity and excessive CD38/CD157 or PARP activation drive NAD^+^ depletion. This metabolic collapse impairs mitochondrial function, enhances oxidative stress, and promotes RGC and RPE damage. The lower panel illustrates downstream NAD^+^-dependent processes: PARP1/2 overactivation consumes NAD^+^ during DNA repair, while reduced SIRT1–7 activity weakens mitochondrial clearance, antioxidant defense, and anti-inflammatory signaling. SIRT1 specifically represses NF-κB, p53, and PRMT1 and promotes vascular and mitochondrial homeostasis *via* regulation of VEGF, HIFs, and FOXO1 NAD^+^-consuming enzymes release NAM, which may be recycled through NAMPT and NMNAT or undergo terminal catabolism to 2PY and 4PY followed by renal excretion. Experimental exposure to 4PY, but not 2PY, increased endothelial VCAM-1 expression and leukocyte adhesion. Green elements denote protective or upregulated mechanisms, whereas red elements indicate pathogenic or downregulated processes contributing to retinal degeneration.

Beyond biosynthesis, NAD^+^ homeostasis also depends on enzymatic consumption, recycling, and terminal nicotinamide catabolism. NAD^+^-cleaving enzymes release nicotinamide (NAM), which may either be recycled through the NAMPT-dependent salvage pathway or methylated to N^1^-methylnicotinamide and subsequently oxidized to the terminal metabolites N^1^-methyl-2-pyridone-5-carboxamide (2PY) and N^1^-methyl-4-pyridone-3-carboxamide (4PY) for renal excretion. Ferrell et al. reported that higher circulating 2PY and 4PY concentrations were associated with cardiovascular events and soluble VCAM-1, while direct exposure to 4PY—but not 2PY—increased VCAM-1 expression in human vascular endothelial cells and promoted endothelial VCAM-1 expression and leukocyte adhesion in mice. These findings suggest that expansion of the niacin/NAD^+^ pool may increase terminal catabolic flux and create a potential vascular-inflammatory safety signal. This mechanism may be relevant to vascular retinal diseases such as diabetic retinopathy and neovascular AMD; however, retinal 4PY accumulation, ocular vascular effects, and a precursor-dose threshold have not been established. The 4PY–VCAM-1 axis should therefore be considered a hypothesis-generating safety constraint rather than a demonstrated cause of retinal injury (Ferrell et al., 2024).

### 3.1 Glaucoma

Retinal ganglion cells (RGCs) operate on a narrow metabolic margin and depend on continuous NAD^+^-supported mitochondrial function to maintain axonal transport and synaptic signaling. Elevated IOP imposes mechanical strain at the optic nerve head, disrupting axoplasmic flow and initiating mitochondrial dysfunction and apoptosis. Concurrently, glutamate-driven excitotoxicity induces calcium overload and oxidative injury, compounding metabolic stress and accelerating neurodegeneration (Cui et al., 2025; Hui, 2026; Lee and Mackey, 2022; Shen et al., 2025; Wang et al., 2025).

Growing evidence reframes glaucoma as not only a pressure-mediated disorder but also a systemic metabolic disease. Patients exhibit reduced mitochondrial activity and diminished circulating NAD^+^ levels, indicating impaired cellular energetics that predispose RGCs to injury identified the NAD^+^/NADH redox ratio as a determinant of RGC resilience (Hui, 2026; Petriti et al., 2021). while Chiarugi (2023) demonstrated that an elevated NMN/NAD^+^ ratio activates a programmed axonal degeneration (PAD) pathway. Together, these findings position NAD^+^ dysregulation as a mechanistic driver of RGC failure (Chiarugi, 2023).

SARM1 (sterile alpha and TIR motif-containing protein 1) is the central executioner of programmed axonal degeneration. Loss of axonal NMNAT2 increases the NMN/NAD^+^ ratio, activating SARM1’s TIR-domain NADase, which rapidly depletes NAD^+^ and triggers metabolic collapse and axonal fragmentation (Essuman et al., 2017; Figley et al., 2021).

Local metabolic constraints further exacerbate vulnerability. The lamina cribrosa limits nutrient delivery, mitochondrial trafficking, and NMNAT2 transport to distal axons, restricting local NAD^+^ synthesis. Human donor retinae confirm this metabolic collapse: although RGCs normally express high levels of NAD^+^-salvage enzymes, glaucomatous eyes show marked NMNAT2 loss, consistent with PAD activation (Tribble et al., 2021).

Beyond neurons, metabolic dysfunction extends to the trabecular meshwork (TM). Liu et al. (2024) showed that NAD^+^ supplementation restores mitochondrial function in glaucomatous TM cells by normalizing cytoskeletal organization, reducing extracellular matrix accumulation, and improving oxidative and phagocytic activity (Liu et al., 2024). These effects were linked to suppression of CD38 and PARP1—major NAD^+^-consuming enzymes—suggesting that NAD^+^ restoration may also improve aqueous outflow and support IOP regulation (Liu et al., 2024).

#### 3.1.1 Preclinical evidence

In the DBA/2J mouse model of glaucoma, NAM supplementation provided robust, dose-dependent neuroprotection. Williams et al. demonstrated that NAM alone preserved RGC structure and function in up to 70% of eyes at lower doses and 93% at higher doses, independent of IOP reduction, confirming a direct NAD^+^-mediated neuroprotective mechanism. In a complementary study, combining NAM with the Wallerian degeneration slow (WldS) allele, which maintains axonal NAD^+^ synthesis, achieved 94% protection against glaucomatous degeneration. This synergistic intervention preserved somal, synaptic, and axonal compartments, maintained anterograde axoplasmic transport, and sustained visual function as assessed by pattern electroretinography (ERG) (Williams et al., 2017a; Williams et al., 2017b).

#### 3.1.2 Clinical evidence

Clinical evidence increasingly links systemic NAD^+^ status to glaucoma susceptibility and treatment response. Nzoughet et al. (2019) demonstrated that patients with POAG have significantly reduced plasma NAM levels, suggesting impaired NAD^+^ metabolism as a systemic vulnerability (Nzoughet et al., 2019). This aligns with findings from the NHANES 2005–2008 cohort (n = 5,780), where higher dietary niacin intake (∼28 mg/day) was associated with a lower risk of developing glaucoma, reinforcing the concept that insufficient NAD^+^ availability may predispose the optic nerve to degeneration (Taechameekietichai et al., 2021).

Interventional studies further support the therapeutic potential of NAD^+^ repletion. Hui et al. (2020) showed that oral NAM (1.5–3.0 g/day) improved retinal function, including significant enhancement of the photopic negative response (PhNR) and visual field mean deviation (Hui et al., 2020). Building on this, (De Moraes et al., 2022), conducted a phase 2 randomized, double-blind, placebo-controlled trial in POAG patients using ascending doses of NAM (1,000–3,000 mg/day) combined with pyruvate (1,500–3,000 mg/day) (De Moraes et al., 2022). The treatment significantly increased the number of improving visual field locations and improved pattern standard deviation, while remaining safe and well tolerated; providing the first randomized evidence that metabolic co-supplementation can yield short-term functional improvements. These short-term functional improvements may represent neuroenhancement or improved neuronal performance and should not be interpreted as evidence of clinical neuroprotection or disease modification, which would require longitudinal demonstration of slower structural or functional progression.

Next-generation NAD^+^ precursors are also under investigation. NR), which has higher systemic bioavailability than NAM, is currently being evaluated in a multicenter randomized trial (NR 300 mg/day for 24 months) with retinal nerve fiber layer (RNFL) thinning as the primary endpoint (Leung et al., 2022). Early exploratory data suggest that NR may modestly improve retinal perfusion density in both glaucoma patients and healthy individuals, as measured by OCT angiography (Gustavsson et al., 2023). However, achieving adequate systemic levels does not necessarily guarantee sufficient intraocular bioavailability due to blood–ocular barriers, a factor that may partly explain inconsistent outcomes in nutritional intervention trials. Moreover, limitations remain as trials are short-term, sample sizes are small, and structural outcomes (OCT RNFL thinning, visual field progression) are not yet proven. Thus, while nicotinamide is one of the most biologically plausible neuroprotective supplements in glaucoma, it remains adjunctive and investigational, not standard of care (Chaudhry et al., 2022).

### 3.2 Diabetic retinopathy

DR is driven by chronic hyperglycemia, which rapidly depletes intracellular NAD^+^, disrupts redox balance, and impairs mitochondrial function in retinal neurons, endothelial cells, pericytes, and RPE. This early metabolic failure heightens susceptibility to oxidative stress and apoptosis, establishing NAD^+^ decline as a central initiating event in DR pathogenesis (Kang and Yang, 2020; Miller et al., 2020). Excessive substrate flux and lactate accumulation further shift the NAD^+^/NADH ratio, exacerbating mitochondrial dysfunction and metabolic inflexibility (Fan et al., 2020; He et al., 2025; Seo et al., 2025).

Distinct metabolic defects characterize type 1 and type 2 diabetes. In type 1 diabetes, oxidative DNA damage drives PARP overactivation, accelerating NAD^+^ consumption and precipitating energetic collapse. In type 2 diabetes, AMPK suppression reduces mitochondrial biogenesis and downregulates NAMPT and PRPP synthesis, impairing the NAD^+^ salvage pathway. Experimental knockdown of NMNAT1 or NAMPT similarly worsens neuronal and vascular injury, underscoring the retina’s dependence on sustained NAD^+^ biosynthesis (Fan et al., 2020; Jadeja et al., 2020; Miller et al., 2020). Collectively, DR is now recognized as both a neurodegenerative and microvascular disease in which neuronal dysfunction emerges before overt vascular regression (He et al., 2025; Seo et al., 2025).

Hyperglycemia triggers multiple damaging pathways, including polyol flux, PKC activation, and accumulation of ROS, that compromise the blood–retina barrier and promote inflammatory cytokine release (TNF-α, IL-1β, VEGF). These cascades inhibit NAD^+^-dependent deacetylases such as SIRT1, SIRT3, and SIRT6, weakening mitochondrial quality control, chromatin regulation, and anti-inflammatory signaling. SIRT1, in particular, supports retinal resilience by repressing pro-inflammatory microRNAs (miR-23b-3p, miR-34a) and downregulating mediators such as NF-κB, p53, VEGF, and HIFs (Kang and Yang, 2020; Karbasforooshan and Karimi, 2018; Li et al., 2023; Lin and Apte, 2018).

Restoring NAD^+^ counteracts these pathological cascades by enhancing oxidative phosphorylation, stabilizing mitochondrial dynamics, reactivating sirtuin-mediated antioxidant defenses, and limiting PARP-driven NAD^+^ loss. NAM supplementation additionally reduces Müller gliosis, dampens neuroinflammation, and preserves retinal structural and functional integrity (Jung et al., 2022; Pu et al., 2022; Yoshino et al., 2018).

#### 3.2.1 Preclinical evidence

Streptozotocin (STZ)-induced diabetic rodent models provide strong experimental evidence that NAD^+^ precursor supplementation protects the diabetic retina. Intraperitoneal or oral administration of NMN or NAM reliably elevates retinal NAD^+^ levels, as confirmed by enzymatic assays and mass spectrometry. This biochemical restoration leads to measurable functional and structural benefits. ERG recordings show preserved retinal responses, while OCT imaging demonstrates reduced thinning and maintenance of retinal layer organization. Importantly, these protective effects are observed across both early (4-week) and later (12-week) stages of experimental diabetes, indicating that NAD^+^ augmentation provides durable and stage-independent retinal protection (Jung et al., 2022).

#### 3.2.2 Clinical evidence

Patients with diabetes exhibit reduced circulating NAD^+^ and impaired salvage pathway activity (NAMPT deficiency), reinforcing the translational rationale for targeting NAD^+^ metabolism as a therapeutic strategy (Fan et al., 2020). Clinically, long-term supplementation with B vitamins (including B3) has been associated with slower development of DR and reduced oxidative stress markers in patients with type 2 diabetes (Pramanik et al., 2022). However, these studies typically involve vitamin combinations, making it difficult to isolate the specific contribution of NAD^+^ repletion. Ongoing trials aim to determine whether NAD^+^ augmentation can slow or prevent diabetic neurodegeneration.

### 3.3 AMD

Mitochondrial dysfunction is a defining feature of both geographic atrophy and neovascular AMD. Chronic oxidative stress, lipid peroxidation, and impaired mitochondrial quality control progressively deplete NAD^+^, reducing OXPHOS efficiency and increasing ROS generation (Jadeja et al., 2020; Lin and Apte, 2018).

Age-related decline in the NAD^+^ salvage pathway, primarily due to reduced NAMPT activity, further limits NAD^+^ biosynthesis. Experimental NAMPT inhibition (e.g., FK866) reproduces these defects, shifting cellular metabolism toward glycolysis and suppressing mitochondrial biogenesis regulators such as PGC-1α, NRF1, and TFAM. This metabolic reprogramming promotes RPE senescence, a hallmark of AMD progression (Jadeja et al., 2020).

Oxidative DNA damage additionally triggers PARP hyperactivation, accelerating NAD^+^ consumption and ATP loss while suppressing sirtuin activity (Lin and Apte, 2018). Because SIRT1 relies on NAD^+^, its inactivation disrupts the PGC-1α–SIRT1 regulatory axis, impairing UPRmt signaling, mitophagy, and mitochondrial maintenance. Restoring NAD^+^ reactivates SIRT1, enhances autophagic flux, engages PINK1/PARKIN pathways, and preserves mitochondrial integrity. SIRT1 also deacetylates autophagy proteins (Atg5, Atg7, Atg8, ULK1) and modulates epigenetic regulators (↑DNMT1, ↓TET1), supporting anti-inflammatory and anti-senescence defenses in the RPE (Cimaglia et al., 2020; Saini et al., 2017).

#### 3.3.1 Preclinical evidence

In rodent light-induced retinal damage (LIRD), photoreceptor and RPE NAD^+^ levels decline sharply in parallel with oxidative stress, mitochondrial dysfunction, and cell death. Supplementation with NAD^+^ precursors such as NAM or NR replenish retinal NAD^+^ pools and significantly reduces photoreceptor loss, preserves RPE integrity, and improves retinal function (Ji et al., 2008). In LIRD, NR additionally promotes mitochondrial biogenesis, limits apoptosis, and suppresses microglial activation; these effects mirror the protection achieved by pharmacologic inhibition of STING (H151), implicating suppression of DNA damage–induced innate immune signaling and pyroptosis as a key neuroprotective mechanism (Zhu S. et al., 2025).

Genetic and pharmacologic augmentation of NAD^+^ biosynthesis supports these findings. Overexpression of NMNAT1 mitigates ROS accumulation, prevents PARP1 hyperactivation, and enhances autophagic flux, while PARP inhibition with olaparib similarly preserves retinal morphology and attenuates oxidative and inflammatory signaling by limiting pathological NAD^+^ consumption (Jadeja et al., 2020; Kovacs et al., 2019). Chronic NAM administration in aged mice maintains rod function and outer retinal structure in a dose-dependent manner, indicating durable efficacy across aging-related metabolic decline (Bassi et al., 2024).

Human cell-based systems corroborate these protective mechanisms. In iPSC-derived RPE from AMD patients and controls, NAM restores mitochondrial respiration, normalizes complement and inflammatory mediators (C3, CFB, CXCL12, ICAM1), and reduces secretion of AMD-linked proteins (APOE, APOJ, VEGF-A, Aβ-42). Multi-omics profiling demonstrates coordinated activation of SIRT1, improved oxidative metabolism, and induction of chromatin-stabilizing anti-senescence programs (Saini et al., 2017).

Complementary studies in ARPE-19 and primary human RPE show NAM-driven increases in mitochondrial size, cristae density, and respiratory efficiency—effects dependent on intact mitochondrial metabolism and reversible upon NAD^+^ withdrawal (Hazim et al., 2022). NAM also synergizes with rapamycin to preserve RPE survival and mitochondrial quality control (Cimaglia et al., 2020).

#### 3.3.2 Clinical evidence

Clinical trials directly evaluating NAD^+^ precursors in AMD are still lacking; however, large-scale epidemiologic and metabolomic studies offer important indirect evidence linking NAD^+^ metabolism to disease susceptibility and progression. Observational human data provide indirect support. In the Age-Related Eye Disease Study cohort, higher dietary intake of niacin and other B vitamins was associated with reduced risk of progression to geographic atrophy in some analyses, although effects were modest and often genotype-dependent (Merle et al., 2016). In a *post hoc* analysis of the AREDS and AREDS2 cohorts showed that higher dietary intake of several nutrients, including vitamins, minerals, and carotenoids, was associated with a reduced risk of progression to late AMD, particularly geographic atrophy (GA). Notably, dietary niacin (vitamin B3) showed only a nominal, non-significant association with AMD risk in this analysis (Chew et al., 2013; Keenan et al., 2020; Vitale et al., 2020).

More recent studies, however, suggest that impaired NAD^+^ availability may still be relevant. A Czech case–control study reported that AMD patients consume significantly less dietary energy and niacin than controls, and metabolomic profiling has similarly identified reduced NAM levels in both vitreous and plasma of intermediate AMD patients, supporting systemic NAD^+^ insufficiency as a potential metabolic biomarker of disease (Bassi et al., 2024). These findings align with complementary cross-sectional analyses showing lower niacin intake among individuals with AMD compared with age-matched controls (Jadeja et al., 2020). However, direct interventional trials of NAD^+^-boosting supplements (NR, NMN, or high-dose NAM) in AMD are currently lacking. This stands in contrast to the enthusiasm seen in aging research and highlights a major evidence gap. Importantly, NAD^+^ modulation has not yet demonstrated structural slowing of GA or drusen regression, unlike the robust functional signals seen in glaucoma.

### 3.4 Safety and guidelines

NAD^+^ precursors are generally well tolerated at standard doses. NR is well tolerated up to 2000 mg/day over 8–12 weeks and up to 3,000 mg/day in short-term safety trials, with only mild or transient adverse effects observed at higher doses (Berven et al., 2023; Martens et al., 2018; Trammell et al., 2016). NMN has been used in randomized clinical trials with no major adverse up to 900 mg/day for 60 days (Mills et al., 2016; Okabe et al., 2022). NAM has been extensively used in clinical and experimental studies. It is generally well tolerated at doses up to 3000 mg/day in chronic use, with only mild gastrointestinal side effects reported (Cimaglia et al., 2020).

Pharmacological doses of NAM (≥3 g/day) raise safety concerns. Two cases of drug-induced liver injury (DILI) have been reported at 3 g/day. The American glaucoma Society (AGS) and the American Academy of Ophthalmology (AAO) issued a joint position statement (Shukla et al., 2025). The statement emphasizes that NAM is not FDA-approved for glaucoma and that high doses (≥3 g/day) should be restricted to controlled clinical trials. If lower doses are prescribed, regular liver function monitoring is strongly advised to mitigate potential hepatotoxicity. The organizations further caution that niacin (nicotinic acid) should not be substituted for NAM because of its higher risk of liver toxicity and other adverse effects.

In diabetic contexts, high-dose NAM or its metabolites may generate toxic catabolites that exacerbate oxidative stress or impair β-cell function. Co-administration with insulin sensitizers, polyphenols, benfotiamine, acetyl-L-carnitine, or aldose reductase inhibitors has been proposed to enhance benefit and minimize potential risk (Fan et al., 2020). Monitoring serum NAD^+^ levels and urinary NAM metabolites may improve dosing precision and safety in metabolic disease populations. For trials using pharmacological doses of NAD^+^ precursors, the relationships among precursor dose, renal clearance, circulating or urinary 2PY/4PY concentrations, and vascular-inflammatory markers warrant prospective investigation. Such metabolic monitoring may help define exposure–response relationships, but it remains investigational, and no validated 4PY threshold for retinal or systemic toxicity is currently available (Ferrell et al., 2024).

## 4 Citicoline

Citicoline (cytidine diphosphate-choline) is a naturally occurring endogenous mononucleotide composed of ribose, cytosine, pyrophosphate, and choline. Although humans can synthesize choline, endogenous production is insufficient to meet metabolic requirements, and supplementation is often necessary. Citicoline is currently available as an intramuscular injection, oral formulation, and topical solution, to be instilled as eye drops. After oral administration, it is hydrolyzed to form choline and cytidine, which are used in the Kennedy cycle to generate phospholipids (García-López et al., 2023).

The Kennedy pathway is not energetically neutral. Choline kinase consumes ATP to generate phosphocholine, whereas (cytidine triphosphate) CTP cytidylyltransferase uses CTP to form CDP-choline. In humans, citicoline-derived uridine is converted to UTP and CTP through ATP-dependent nucleotide-salvage reactions. Thus, citicoline supplies membrane precursors but does not bypass cellular bioenergetic requirements; whether this demand limits efficacy or imposes a meaningful energetic burden in the metabolically compromised aging retina remains unknown (Wurtman et al., 2000; Ross et al., 1997; Fagone and Jackowski, 2013).

Citicoline (CDP-choline) has a substantial translational and clinical evidence base, particularly in neurodegenerative conditions. Outside ophthalmology, citicoline has been extensively studied in stroke, traumatic brain injury, and cognitive impairment, where it supports phospholipid membrane synthesis, mitochondrial stability, and neurotransmitter regulation (Bermejo et al., 2023; Bonvicini et al., 2023; Oddone et al., 2021; Secades et al., 2023). Figure 4 illustrates the potential mechanisms underlying the neuroprotective effects of citicoline.

**FIGURE 4:**
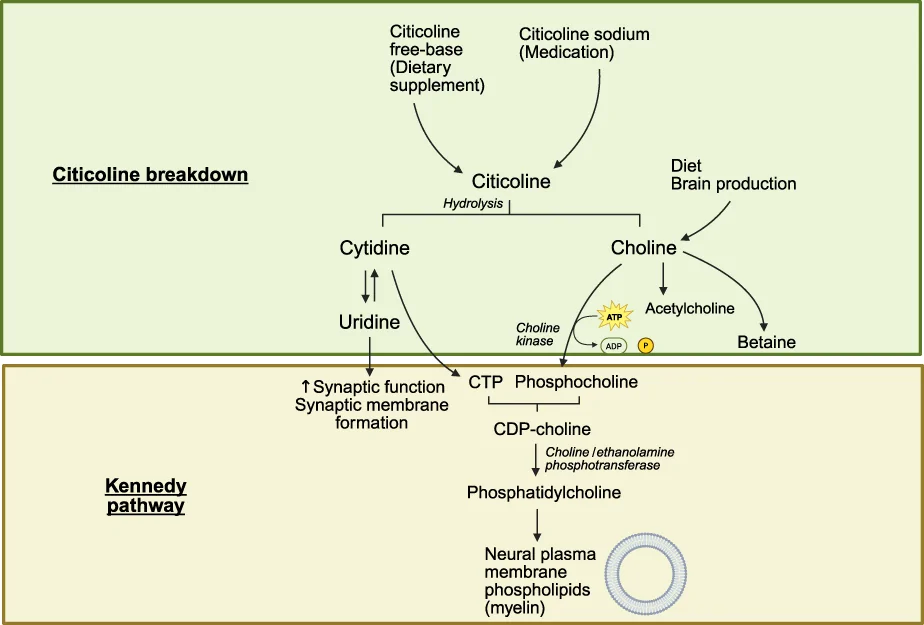
Overview of citicoline metabolism following ingestion. In the bloodstream, citicoline undergoes hydrolysis and dephosphorylation, producing choline and cytidine (converted to uridine in humans), which cross the blood–brain barrier. Within the brain, cytidine is phosphorylated to form cytidine triphosphate (CTP). Choline follows three main pathways: phosphorylation to phosphocholine, oxidation to betaine (N,N,N-trimethylglycine), or conversion to acetylcholine, a neurotransmitter. CTP and phosphocholine subsequently combine to generate CDP-choline, which serves as a precursor for phosphatidylcholine, essential for membrane integrity and myelin metabolism.

In glaucoma, citicoline is one of the most consistently studied neuro-supportive agents, with randomized and longitudinal studies demonstrating improvements in pattern electroretinogram (PERG), visual evoked potentials (VEP), and slower visual field deterioration, independent of intraocular pressure control (Prinz et al., 2023; Study Details NCT05527106, 2026; Study Details NCT06355765, 2026).

However, despite its strong neuroprotective profile, citicoline has not been convincingly studied in AMD or DR. There are no robust clinical trials demonstrating efficacy in photoreceptor degeneration, macular atrophy, or microvascular diabetic retinal disease. Therefore, citicoline currently occupies a disease-specific role, with clinically supported benefits in glaucoma, but insufficient evidence for AMD or DR, highlighting a gap between generalized neuroprotection and disease-specific retinal pathology (Oddone et al., 2021).

### 4.1 Glaucoma

(Faiq et al., 2019; Oddone et al., 2021) Choline contributes to neuronal membrane homeostasis primarily through the Kennedy pathway, in which choline is phosphorylated to phosphocholine and subsequently incorporated into CDP-choline and phosphatidylcholine, a major membrane phospholipid (Secades and Gareri, 2022). Citicoline can therefore support phosphatidylcholine synthesis and membrane repair by providing choline and cytidine-derived intermediates. Choline can also serve as a precursor for acetylcholine, a neurotransmitter, but acetylcholine itself is not a membrane phospholipid. Citicoline has additionally been associated with antioxidant effects, including preservation of glutathione-related defenses, although glutathione is not a direct metabolic product of choline. These mechanisms may contribute to improved neuronal membrane stability and resistance to oxidative injury (Faiq et al., 2019; Oddone et al., 2021).

A 3-year randomized, double-blind trial involving 78 patients with open-angle glaucoma and controlled IOP (<18 mmHg) compared topical citicoline with placebo. Citicoline-treated patients showed significantly slower progression in 10–2 visual-field mean deviation and RNFL thinning, whereas the difference in 24–2 mean-deviation progression was not statistically significant (Rossetti et al., 2020). In another study of OAG patients (IOP <18 mmHg with beta-blocker monotherapy only), 24 eyes received additional topical citicoline (3 drops/day for 4 months) while 23 eyes remained on betablockers alone. Citicoline-treated eyes showed a significant increase in pattern electroretinogram P50N95 amplitude and greater visual evoked potential N75P100 amplitude, and shorter P100 implicit times (all p < 0.01), with changes correlating between PERG and VEP measures (Parisi et al., 2015). Robeti et al. confirmed the improved electrophysiological response after administration of citicoline (2%) eye drops in 16 patients in a study of 36 OAG patients (Roberti et al., 2014).

Oral citicoline has also shown benefit. In a prospective, 600 mg/day citicoline for minimum 3 months in 22 patients improved visual evoked potential amplitude and latency while RNFL thickness remained stable (Chițu et al., 2019). In another randomized study of 60 OAG patients with controlled IOP (≤18 mmHg), a daily oral dose of 500 mg citicoline stabilized visual field indices and preserved RNFL and GCL thickness over 24 months compared to untreated patients, whose measurements declined. The SAP values at 18 months were significantly higher in the citicoline-treated group and their RNFL and GCL thicknesses were significantly greater (Lanza et al., 2019). Furthermore, a study of 109 OAG patients with controlled IOP (<18 mmHg) showed that daily oral supplementation with a fixed combination of citicoline 500 mg, homotaurine 50 mg, and vitamin E 12 mg alongside topical treatment significantly improved contrast sensitivity and quality of life (Marino et al., 2020).

A double-blind study included 29 open-angle glaucoma patients, with 15 in the citicoline group and 14 in the placebo group. After 12 months, the citicoline group showed significant improvements in retinal function and neural conduction (PERG, VEP, RCT), which were associated with visual field changes, suggesting enhanced visual pathway connectivity through neuromodulatory effects (Parisi et al., 2026). A multicenter, randomized, double-masked, placebo-controlled crossover study evaluated citicoline (500 mg/day) in patients with open-angle glaucoma across five European clinics. Citicoline significantly improved vision-related quality of life (VFQ-25 scores), particularly in patients with worse baseline impairment, representing the first placebo-controlled evidence of a treatment enhancing quality of life in glaucoma (Rossetti et al., 2023).

### 4.2 Diabetic retinopathy

Preclinical studies demonstrate complementary mechanisms. Bogdanov reported a study on three groups of mice: control group without diabetes, untreated diabetics, and diabetics treated with citicoline in liposomal solution (2% citicoline) administered topically twice a day for 15 days (Bogdanov et al., 2018). The diabetic animals treated with citicoline presented improvements in ERG B-wave amplitude. GFAP (Glial fibrillary acidic protein) expression was primarily localized to the retinal GCL in non-diabetic control mice (db/+). In contrast, age-matched diabetic mice (db/db) treated with vehicle exhibited significantly increased GFAP expression. Notably, all diabetic mice showed a GFAP score of ≥3. Treatment with citicoline significantly reduced reactive gliosis, with all cases showing a GFAP score of ≤3. The percentage of apoptotic cells in the retinal layers (ONL, INL, and GCL) was significantly increased in diabetic mice compared to age-matched non-diabetic controls. Diabetic mice treated with liposomal citicoline showed a reduced apoptosis rate in the ONL compared to those treated with vehicle. Furthermore, activated caspase three levels were significantly lower in db/db mice receiving citicoline *versus* those receiving vehicle (Bogdanov et al., 2018).

In a 3-year clinical study of patients with type 1 diabetes and mild non-proliferative diabetic retinopathy (NDPR), citicoline (2%) plus vitamin B12 is topically administered to 20 patients. This treatment reduced microvascular damage and neuro-retinal degeneration compared to the untreated control group (Parravano et al., 2020). A similar study involving 21 patients with type 1 diabetes and mild NPDR showed an improved electrophysiological response in those patients treated with citicoline plus vitamin B12 (Parisi et al., 2021). In a randomized placebo-controlled trial of 30 patients with diabetic neuropathy, citicoline plus vitamin B12 improved corneal nerve density and sensitivity. In this study, twenty patients received eye drops three times daily for one and a half years, while ten patients were assigned to the placebo group (Fogagnolo et al., 2020).

This prospective observational study evaluated citicoline eye drops in over 100 patients with nonproliferative diabetic retinopathy. Treatment was associated with reduction or disappearance of macular hard exudates in a small subset of patients, suggesting a potential neuroprotective and clinical benefit, although larger and controlled studies are needed for confirmation (Tomić et al., 2025).

### 4.3 AMD

In an *in-vitro* study, citicoline treatment at a concentration of 1 mM on transmitochondrial AMD RPE cybrid cells resulted in decreased apoptotic cell death, improved cell viability, and reduced oxidative stress. Flow cytometry analysis revealed lower levels of apoptotic markers annexin V and propidium iodide in the citicoline-treated cells. Furthermore, these cells exhibited significantly reduced ROS, indicating a decrease in oxidative stress. Additionally, citicoline-treated cybrids showed reduced expression of HIF-1α (hypoxia-inducible factor 1-alpha) and VEGF genes, both of which are associated with angiogenesis in choroidal neovascularization in AMD (Nashine and Kenney, 2020).

## 5 Pyruvate

Pyruvate is a central glycolytic intermediate that feeds into the tricarboxylic acid cycle and supports oxidative phosphorylation and ATP generation in retinal ganglion cells. These neurons have sustained firing demands and long axons that increase energy requirements (Liang et al., 2025).

The proposed neuroprotective mechanisms of pyruvate are summarized in the left panel of Figure 5. Pyruvate (including sodium pyruvate) has a well-established biochemical and neuro-metabolic rationale but remains largely preclinical in ophthalmology. As a central metabolite of glycolysis and mitochondrial respiration, pyruvate can act as an alternative energy substrate, buffer oxidative stress, and stabilize mitochondrial redox balance. In non-ocular systems, pyruvate has shown neuroprotective effects in models of cerebral ischemia, traumatic brain injury, and mitochondrial dysfunction, where it preserves ATP levels and reduces oxidative damage (Liang et al., 2025).

**FIGURE 5:**
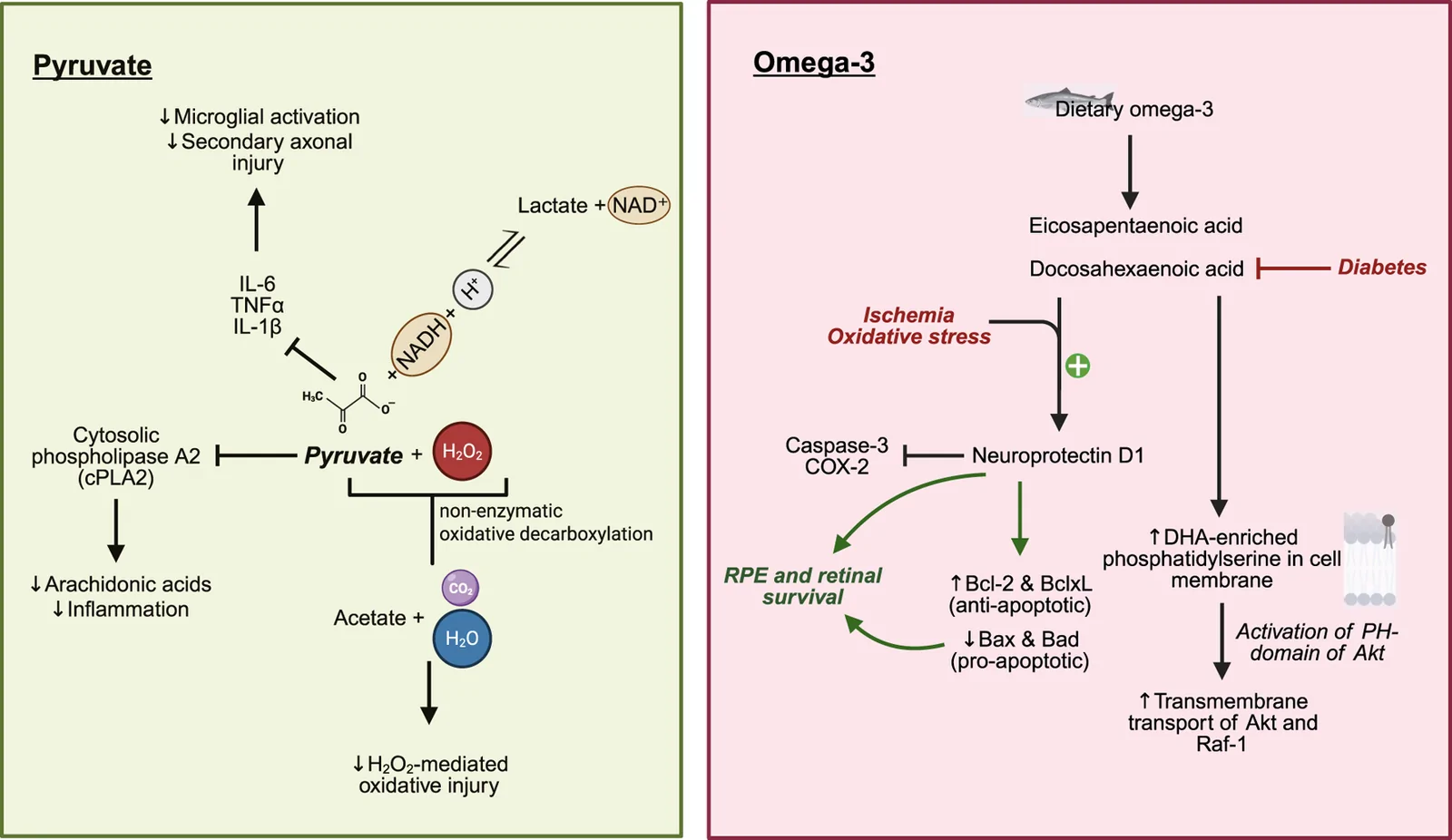
Pyruvate (left panel) undergoes reversible LDH-dependent coupling with lactate and the NADH/NAD^+^ redox pair and nonenzymatically scavenges H_2_O_2_ to produce acetate, CO_2_, and H_2_O. The panel also summarizes its reported effects on cPLA_2_-associated inflammatory signaling, microglial activation, and axonal injury. Omega-3 (right panel) illustrates diabetes-associated retinal DHA depletion, DHA-derived neuroprotectin D1 signaling, inhibition of caspase-3 and COX-2, regulation of Bcl-2 family proteins, and phosphatidylserine-dependent recruitment of Akt and Raf-1, supporting RPE and retinal survival.

In ocular research, current evidence for pyruvate is mostly limited to experimental models, in which pyruvate has been shown to protect retinal ganglion cells against excitotoxicity and ischemic injury (Li K. et al., 2025; Schanzer et al., 2025).A small number of clinical trials related to glaucoma have been registered (Study Details NCT05695027, 2026; Study Details NCT03797469, 2026). However, no randomized clinical trials have evaluated pyruvate supplementation in AMD or DR. Moreover, there are no observational cohort studies, biomarker-driven clinical investigations, or long-term safety and efficacy data in retinal disease populations. Consequently, despite its biological plausibility as a metabolic neuroprotective agent, pyruvate remains an experimental candidate without clinical validation in ocular diseases, including AMD and DR.

### 5.1 Glaucoma

Combination of oral NAM and pyruvate has shown short-term functional benefits in open-angle glaucoma (Study Details NCT03797469, 2026). In a randomized, double-blind phase 2 trial that used ascending doses of NAM at 1,000 to 3,000 mg per day and pyruvate at 1,500 to 3,000 mg per day, participants receiving active treatment had more visual-field locations that improved beyond test–retest variability and exhibited favorable changes in pattern standard deviation over roughly 2 months, with no serious adverse events reported (De Moraes et al., 2022). A multicenter, longer-duration randomized trial is underway to evaluate durability and safety and is designed to capture both functional outcomes and structural measures using optic nerve imaging (Harder et al., 2020). A public registry synopsis reports a target enrollment close to 188 and extended treatment duration to assess longer-term efficacy (Ferrell et al., 2024).

In experimental, early mitochondrial stress and impaired bioenergetic control precede frank degeneration and heighten vulnerability to pressure-related and vascular insults (Muench et al., 2021). Oral pyruvate supplementation restores bioenergetic tone by increasing mitochondrial substrate availability and stabilizing the phosphorylation potential (Muench et al., 2021; Yang et al., 2024). This maintains ATP levels and supports ion pump activity required for action-potential propagation and axonal transport (Muench et al., 2021; Yang et al., 2024). Disrupted glucose and pyruvate handling has been observed in preclinical glaucoma models and includes reduced glycolytic throughput, stress-related inhibition of pyruvate dehydrogenase, and a shift toward less efficient anaerobic metabolism (Desagher et al., 1997; Yang et al., 2024). Exogenous pyruvate may bypass upstream glycolytic limitations, but its metabolic fate is not exclusively mitochondrial. Entry into mitochondrial oxidation depends on transport through the mitochondrial pyruvate carrier, pyruvate dehydrogenase activity, and available respiratory capacity. Cytosolic pyruvate is also coupled to lactate through the near-equilibrium lactate dehydrogenase reaction: pyruvate + NADH + H^+^ ⇌ lactate + NAD^+^. Increasing pyruvate availability can favor rightward flux, oxidizing cytosolic NADH and regenerating NAD^+^, which may sustain glycolysis and alleviate an excessively reduced redox state. However, net flux depends on the lactate-to-pyruvate ratio, NADH/NAD^+^ balance, pH, LDH isoform distribution, mitochondrial utilization, and monocarboxylate transport. If mitochondrial oxidation or lactate export is constrained, lactate accumulation and disruption of cytosolic redox balance are plausible. Importantly, reduction of pyruvate to lactate consumes rather than generates H^+^; therefore, lactate formation should not be described as directly causing intracellular acidification. Any associated acid–base disturbance would instead depend on cellular proton production, buffering, ATP turnover, and proton-coupled monocarboxylate transport. Retinal lactate accumulation or pH toxicity has not been demonstrated at the oral doses used in glaucoma trials (Hegde et al., 2010; Cheng et al., 2019).

Mechanistically, pyruvate contributes to redox buffering through complementary actions. It can directly react with hydrogen peroxide through non-enzymatic decarboxylation to acetate and carbon dioxide, which lowers oxidant burden and limits damage to proteins and lipids (Muench et al., 2021; Yang et al., 2024). By stabilizing mitochondrial respiration, pyruvate can also reduce electron leak that generates superoxide and downstream peroxides, which lessens oxidative pressure on glycolysis and the electron transport chain (Muench et al., 2021; Yang et al., 2024).

During metabolic and oxidative stress, excessive activation of poly (ADP-ribose) polymerase depletes NAD^+^ and impairs glyceraldehyde-3-phosphate dehydrogenase, which depresses glycolysis and lowers ATP availability. Experimental work in neurons and retina links PARP overactivation and NAD^+^ depletion to cell-death pathways relevant to glaucoma. Maintaining redox homeostasis and metabolic flux limits PARP engagement and preserves the intracellular NAD^+^ pool (Alano et al., 2010; Petriti et al., 2021). Co-administration of NAM augments NAD^+^ biosynthesis through the salvage pathway and complements pyruvate’s metabolic support in a mechanistically coherent manner (Harder et al., 2020; Tribble et al., 2021).

Pyruvate is also associated with reduced microglial activation and lower expression of inflammatory mediators in ocular-hypertension paradigms, which reduces secondary axonal injury and preserves synaptic function. Improved cellular energy status can reduce glutamatergic stress and stabilize calcium handling by maintaining ATP-dependent ion pumps and buffering systems that prevent excitotoxic cascades (Muench et al., 2021; Yang et al., 2024). RGCs express the mitochondrial pyruvate carrier, which enables mitochondrial access to exogenous pyruvate. *In vivo* supplementation increases intra-retinal pyruvate, supporting the interpretation that administered substrate reaches the target tissue and engages the intended metabolic pathway (Grenell et al., 2019; Harder et al., 2020; Do Rhee et al., 2022).

Across rat and mouse ocular-hypertension models, pyruvate administered alone or with NAM preserved ganglion-cell somas and axons and improved functional readouts that include pattern electroretinography and optomotor behavior. These studies also showed reduced activation of stress-response pathways triggered by pressure and ischemia. Independent manipulations of nutrient-sensing networks, including mTOR and autophagy, further support a metabolic nexus for protection in which adequate substrate supply and balanced growth signaling determine survival under glaucoma-relevant stress (Huang et al., 2024; Muench et al., 2021; Zhu et al., 2025a).

Human metabolomics provide convergent support. Analyses that leverage large biobanks report that higher circulating levels of pyruvate-related energetic metabolites associate with lower glauoma risk and that these signatures offer modest incremental value over polygenic risk scores in prediction models. These findings are consistent with the concept that energetic resilience modulates disease susceptibility at the population level (Li et al., 2025b). They also complement population-scale metabolomic work that links systemic energy metabolism with glaucoma phenotypes across multiple cohorts including UK Biobank (Zeleznik et al., 2023). Together with the phase 2 clinical signal, these data provide a coherent translational rationale for longer-term evaluation of NAM plus pyruvate, designed to test structural outcomes by optic nerve imaging alongside standard automated perimetry (Muench et al., 2021; Yang et al., 2024).

## 6 Omega-3

Long-chain omega-3 polyunsaturated fatty acids (LC-ω3PUFAs), especially eicosapentaenoic acid (EPA) and docosahexaenoic acid (DHA), are integral components of neuronal membranes, retinal cell membranes and play key roles in photoreceptor integrity, synaptic signaling, inflammation resolution, and vascular homeostasis, with DHA highly enriched in photoreceptors and other retinal layers. These biological properties generated strong expectations that omega-3 supplementation could slow the progression of major retinal diseases. The proposed neuroprotective mechanisms of omega-3 fatty acids are summarized in the right panel of Figure 5.

However, DHA’s redox effects are context dependent. Its six double bonds make DHA-containing phospholipids highly susceptible to nonenzymatic radical peroxidation. In light- and oxidant-stressed retinal and RPE models, DHA oxidation generates lipid hydroperoxides, reactive aldehydes, and carboxyethylpyrrole adducts that can impair lysosomal and mitochondrial function. Conversely, DHA is essential for photoreceptor membranes and supports the regulated formation of cytoprotective mediators such as neuroprotectin D1. The net effect therefore depends on membrane incorporation, oxidative and iron burden, and antioxidant and repair capacity. AREDS2 did not measure retinal DHA incorporation or lipid-peroxidation products; peroxidation should therefore be considered a plausible modifier of response, not an established cause of the null result (Cheng et al., 2019; Liu et al., 2014; Liu et al., 2022).

Accordingly, numerous clinical trials have investigated omega-3 fatty acids in ocular conditions, including glaucoma (Study Details NCT01630551, 2026; Study Details NCT04499820, 2026; Study Details NCT01646047, 2026; Study Details NCT00135226, 2026; Study Details NCT06489873, 2026; Study Details NCT05763940, 2026; Study Details NCT01404845, 2026; Study Details NCT01782352, 2026; Study Details NCT00060749, 2026). However, clinical outcomes have been inconsistent, and omega-3s represent a clear example where strong biological plausibility has not reliably translated into clinical benefit—likely in part because ocular bioavailability and tissue distribution differ substantially between experimental animal models and the human eye.

### 6.1 Diabetic retinopathy

In the diabetic retina, chronic hyperglycemia induces oxidative stress, para-inflammation, and microvascular dysfunction that together drive neurodegeneration and microvascular injury. In preclinical diabetic-retina models*,*LC-ω3PUFAs mitigate these processes by limiting lipid peroxidation, bolstering endogenous antioxidant defenses, and suppressing leukostasis and cytokine signaling, including TNF-α and IL-6, while attenuating VEGF-driven angiogenic pathways and apoptosis in retinal cells (Georgiou and Prokopiou, 2023; Zeppieri et al., 2024). Mechanistically, DHA integrates into phosphatidylserine-rich membrane microdomains and modulates pro-survival kinase cascades such as Akt and Raf-1, thereby promoting neuronal resilience under stress (Akbar et al., 2005; Kim et al., 2010). Enzymatic derivatives of DHA, most notably neuroprotectin D1 (NPD1), exert potent anti-inflammatory and anti-apoptotic actions in human RPE and neural retina (Mukherje et al., 2007; Mukherjee et al., 2004).

Preclinical studies show that diets enriched with EPA and DHA restore retinal DHA depletion induced by diabetes, reduce inflammatory and angiogenic signaling, and improve functional visual readouts (Georgiou and Prokopiou, 2023; Zeppieri et al., 2024).

Large observational cohorts support clinical relevance. In a pre-specified analysis of the PREDIMED population, higher intake of LC-ω3PUFAs, approximately at least 500 mg per day, was associated with a reduced incidence of sight-threatening DR in older adults with type 2 diabetes (Sala-Vila et al., 2016). Independent analyses of dietary fish intake suggest that about two servings of fish per week provide a practical behavioral proxy for achieving LC-ω3PUFA exposure associated with lower DR risk (Chew, 2017). (Georgiou and Prokopiou, 2023). However, these observational associations with dietary omega-3 or fish intake should not be interpreted as evidence of benefit from omega-3 supplementation.

Randomized evidence from ASCEND-Eye did not demonstrate a significant protective effect of omega-3 supplementation against diabetic retinopathy (Lin and Apte, 2018). Similarly, the 2025 ASCEND-Eye analysis did not demonstrate a clear protective effect against AMD, although this analysis was limited by statistical power. Thus, while observational evidence supports an association between dietary omega-3 intake and lower retinal disease risk, current randomized evidence does not establish omega-3 supplementation as an effective strategy for primary prevention or early-stage risk reduction (Georgiou and Prokopiou, 2023).

### 6.2 Glaucoma

Interest in omega-3s for glaucoma as a neuroprotective adjunct stems from potential improvements in ocular perfusion and neuroinflammation. Controlled studies have not demonstrated consistent reductions in intraocular pressure or visual field progression attributable to omega-3 supplementation alone. A randomized clinical study reported that combined oral DHA plus citicoline improved visual-field indices and their slopes after 3 months in patients with glaucoma, although the design does not allow attribution to DHA alone (Anton et al., 2022). Across rodent glaucoma models and retinal cell systems, DHA supplementation reduces oxidative injury, stabilizes mitochondrial function, and limits apoptosis (Lafuente et al., 2021). These effects align with membrane-centric signaling actions of DHA, which enhance membrane fluidity, facilitate recruitment of survival kinases such as Akt and Raf-1, and promote downstream cytoprotective signaling (Akbar et al., 2005; Kim et al., 2010). The biosynthesis of NPD1 from DHA in stressed retinal cells provides an additional pathway for dampening inflammation and countering cell-death program (Mukherje et al., 2007; Mukherjee et al., 2004).

### 6.3 AMD

Early epidemiologic studies suggested that higher dietary intake of fish or omega-3 fatty acids was associated with reduced risk of AMD progression. This led to inclusion of DHA and EPA in the Age-Related Eye Disease Study 2 (AREDS2). In its primary analysis*,* AREDS2 showed no additional benefit of DHA/EPA supplementation (350 mg DHA +650 mg EPA daily) on progression to advanced AMD when added to the standard antioxidant and zinc formulation (Chew et al., 2013). This null result persisted across AMD severity levels, genetic subgroups, and long-term follow-up (Keenan et al., 2020; Querques et al., 2014; Vitale et al., 2020).

The Nutritional AMD Treatment-2 (NAT-2) trial evaluated long-term DHA supplementation (840 mg/day for 3 years) in patients with unilateral neovascular AMD. Detailed *post hoc* analyses showed no significant effect of DHA on drusen number, area, or remodeling, despite prolonged exposure (Querques et al., 2016). Interestingly, drusen regression correlated more strongly with age, sex, and CFH genotype than with DHA intake, reinforcing the concept that omega-3s do not meaningfully alter AMD structural biology once disease is established.

#### 6.3.1 Overall interpretation

Several factors likely explain the discrepancy and ambiguity between biology and outcomes.

- Timing: Omega-3s may be preventive rather than therapeutic; trials enrolled patients with established disease.
- Bioavailability: Retinal incorporation of DHA may be tightly regulated and resistant to supplementation.
- Ceiling effects: Modern diets and baseline intake may already saturate retinal DHA needs.
- Disease complexity: AMD and DR progression are driven by genetics, complement activation, and metabolic dysfunction beyond inflammation alone.
- Efficacy signals with ω3 interventions can vary by chemical form and dose. Differences between ethyl-ester, triglyceride, and re-esterified triglyceride preparations influence bioavailability and may shape ocular tissue exposure.

Although not specific to DR or glaucoma, contemporary randomized data in ophthalmology show that formulation alone does not guarantee superiority overactive comparators, underscoring the need for rigorous dose–exposure–response work in retinal neurodegeneration (Eom et al., 2024). Future trials should incorporate pharmacokinetic and lipidomic endpoints, standardize background diet, and stratify by metabolic control and disease stage to clarify which patients and formulations benefit most.

## 7 Discussion and limitations

### 7.1 Main findings

Across the four interventions reviewed, a consistent mechanistic thread emerges: NAD^+^ boosters, citicoline, pyruvate, and omega-3 fatty acids each target one or more core hallmarks of retinal aging, including mitochondrial bioenergetic decline, redox imbalance, membrane instability, and low-grade neuroinflammation. The maturity of supporting evidence, however, differs substantially across interventions. NAD^+^ boosters and pyruvate are supported primarily by strong mechanistic rationale and encouraging preclinical or early-phase clinical signals, with robust human efficacy data still lacking. Citicoline has accumulated the most extensive clinical trial experience, particularly in glaucoma, although heterogeneous formulations and outcome measures limit direct comparison across studies. Omega-3 fatty acids present the clearest divergence between evidence types: favorable observational associations with dietary intake have not been consistently replicated in randomized supplementation trials, including AREDS2 and ASCEND-Eye. Taken together, the current evidence supports biological plausibility and early functional or biomarker signals for these interventions, particularly in glaucoma and diabetic retinal disease, but does not yet establish disease-modifying efficacy for any of the four. In contrast, the AREDS/AREDS2 formulations remain the best-established nutritional interventions in retinal disease, supported by large randomized trials demonstrating reduced risk of progression to advanced AMD in appropriately selected patients. This distinction highlights the gap between mechanistic or early clinical evidence for the four interventions reviewed here and the level of evidence required for routine clinical recommendation.

### 7.2 Clinical implications

These findings have implications for clinician–patient discussions regarding nutritional and metabolic interventions in retinal disease. At present, none of the four interventions reviewed here has sufficient evidence to support its use as a disease-modifying therapy, and none should be considered a substitute for established standard-of-care treatment for AMD, DR, or glaucoma. Citicoline and omega-3 fatty acids have a longer history of clinical use and more established safety profiles, but their potential benefits should be discussed cautiously in light of heterogeneous or inconclusive efficacy evidence. NAD^+^ boosters and pyruvate remain investigational, and their clinical interpretation should be framed accordingly, particularly given the potential dose-dependent risks discussed above. Importantly, dose, formulation, and route of administration should be considered when interpreting evidence for any of these agents, as findings from pharmacological-dose or non-oral interventions do not necessarily generalize to conventional dietary supplementation. This distinction is particularly important for nicotinamide, for which the high doses investigated in glaucoma trials should not be equated with routine vitamin B3 supplementation and may require clinical monitoring because adverse effects, including hepatotoxicity, have been reported at high doses.

### 7.3 Therapeutic context and terminology

The four interventions differ substantially in their formulation, dose, route of administration, intended use, and regulatory context. Accordingly, the terms dietary supplement, nutraceutical, and therapeutic intervention should not be considered interchangeable. In particular, high-dose nicotinamide investigated experimentally in glaucoma represents a pharmacological-dose intervention and should be distinguished from conventional dietary vitamin supplementation. Similarly, citicoline has been investigated through oral, topical, and intramuscular routes, each with different therapeutic and regulatory contexts. These distinctions are important when translating findings across preclinical studies, clinical trials, and routine nutritional use.

### 7.4 Translational challenges and future directions

Several challenges remain in translating these interventions into clinical practice. Preclinical studies often employ doses, formulations, treatment durations, and disease models that may not directly correspond to human use. Clinical studies also differ substantially in disease stage, baseline nutritional status, concomitant treatments, intervention dose and route, and outcome definitions. Future studies should therefore prioritize adequately powered randomized trials with standardized formulations and clinically meaningful ophthalmic outcomes, while also considering whether treatment effects vary according to disease stage, metabolic status, or baseline nutritional deficiency. Given the multifactorial biology of retinal neurodegeneration, future studies may also investigate rational combination strategies targeting complementary pathways rather than individual metabolic mechanisms in isolation. However, combination approaches introduce additional challenges, including potential interactions, difficulty attributing efficacy or adverse effects to individual components, optimization of dosing and sequencing, and greater regulatory complexity. Accordingly, the potential benefit of combining these interventions remains hypothetical and requires direct experimental and clinical evaluation.

### 7.5 Neuroenhancement *versus* neuroprotection

An important consideration when interpreting the current evidence is the distinction between short-term functional improvement and clinical neuroprotection. Improvements in measures such as PhNR, PERG, VEP, or visual-field sensitivity may indicate functional enhancement or improved neuronal performance but do not, by themselves, demonstrate preservation of neural tissue or modification of disease progression. Evidence of clinical neuroprotection requires longitudinal demonstration of slower structural and/or functional deterioration. This distinction is particularly relevant to the early NAD^+^ and nicotinamide–pyruvate studies, in which encouraging short-term functional signals should be considered hypothesis-generating rather than evidence of established disease-modifying efficacy.

### 7.6 Limitations

This review also has several limitations. Although we performed a structured search of the literature and clinical trial registries, the review was designed around a translational framework linking ocular disease, aging-related mechanisms, and metabolic interventions rather than as a quantitative meta-analysis. The heterogeneity of the included interventions and evidence—including preclinical studies, observational studies, and clinical trials—limits direct comparisons and precludes conclusions regarding comparative efficacy. Furthermore, the four interventions were selected based on their relevance to the proposed metabolic-aging framework and therefore do not represent an exhaustive evaluation of all supplements or neuroprotective interventions investigated for retinal disease. These limitations should be considered when interpreting the proposed framework and its potential clinical implications.

## 8 Conclusion

Retinal neurodegeneration in age-related macular degeneration, diabetic retinopathy, and glaucoma reflects the convergence of disease-specific pathology with fundamental aging-related processes, including mitochondrial dysfunction, metabolic insufficiency, redox imbalance, and chronic low-grade inflammation. While AREDS2 remains the only nutritional intervention supported by large randomized clinical trials showing a clear reduction in progression to advanced AMD, the broader field of metabolic and neuroprotective supplementation is evolving rapidly. In this review, NAD^+^ precursors, citicoline, pyruvate, and omega-3 fatty acids emerged as particularly relevant candidates. These four were selected from a heterogeneous landscape of over 40 identified supplements because they uniquely converge on aging-related biological pathways across all three diseases examined, maintain a coherent translational trajectory from bench to early clinical evidence, and raise clinically actionable questions around dosing, safety, and patient counseling.

This review adopts a conceptual framework in which AMD, DR, and glaucoma may be viewed within the framework of accelerated retinal aging, while recognizing their distinct disease-specific mechanisms, and metabolic supplements are evaluated based on their capacity to modulate aging-related pathways relevant to retinal neurodegeneration. Current evidence supports strong biological plausibility and encouraging preclinical and early clinical signals, particularly in glaucoma and diabetic retinal disease; however, long-term efficacy, optimal dosing, ocular bioavailability, and safety remain insufficiently established. Accordingly, these agents should currently be regarded as investigational or adjunctive rather than definitive therapies, and they should not replace established evidence-based care. At present, their most appropriate clinical role lies in informed clinician–patient discussion, especially in the context of progressive disease and limited therapeutic options. Future studies should prioritize adequately powered, disease-specific randomized trials, improved strategies for ocular tissue delivery, and biomarker-guided approaches that may help identify which patients are most likely to benefit. Overall, metabolic supplementation represents a promising but still developing strategy for modulating pathways implicated in retinal neurodegeneration, with its potential to preserve retinal function or slow disease progression requiring confirmation in longitudinal clinical trials.

## Glossary

AMD: Age-Related Macular Degeneration
AREDS: Age-Related Eye Disease Study
AREDS2: Age-Related Eye Disease Study two
ATP: Adenosine Triphosphate
BCVA: Best-Corrected Visual Acuity
CD38: Cluster of Differentiation 38
CFH: Complement Factor H
CNS: Central Nervous System
COX-2: Cyclooxygenase-2
DR: Diabetic Retinopathy
DME: Diabetic Macular Edema
DHA: Docosahexaenoic Acid
EPA: Eicosapentaenoic Acid
ERG: Electroretinography
GA: Geographic Atrophy
GCL: Ganglion Cell Layer
GCIPL: Ganglion Cell–Inner Plexiform Layer
GFAP: Glial Fibrillary Acidic Protein
GL: Glaucoma
IOP: Intraocular Pressure
LC-ω3PUFA: Long-Chain Omega-3 Polyunsaturated Fatty Acid
LIRD: Light-Induced Retinal Damage
MPOD: Macular Pigment Optical Density
**NAD** ^**+**^: Nicotinamide Adenine Dinucleotide
NAM: Nicotinamide
NAMPT: Nicotinamide Phosphoribosyltransferase
NMN: Nicotinamide Mononucleotide
NMNAT: Nicotinamide Mononucleotide Adenylyltransferase
NPDR: Non-Proliferative Diabetic Retinopathy
NPD1: Neuroprotectin D1
OCT: Optical Coherence Tomography
OCT-A: Optical Coherence Tomography Angiography
OAG: Open-Angle Glaucoma
OXPHOS: Oxidative Phosphorylation
PAD: Programmed Axonal Degeneration
PARP: Poly(ADP-ribose) Polymerase
PERG: Pattern Electroretinogram
POAG: Primary Open-Angle Glaucoma
PUFA: Polyunsaturated Fatty Acid
RGC: Retinal Ganglion Cell
RNFL: Retinal Nerve Fiber Layer
ROS: Reactive Oxygen Species
RPE: Retinal Pigment Epithelium
SAP: Standard Automated Perimetry
SIRT: Sirtuin
STZ: Streptozotocin
TM: Trabecular Meshwork
VEGF: Vascular Endothelial Growth Factor
VEP: Visual Evoked Potential
